# Automated machine-learning framework for predicting drug solubility in supercritical CO_2_ for sustainable process development

**DOI:** 10.1038/s41598-026-59449-z

**Published:** 2026-06-25

**Authors:** Saad M. Alshahrani, Mahboubeh Pishnamazi

**Affiliations:** 1 Department of Pharmaceutics, College of Pharmacy, Prince Sattam Bin Abdulaziz University, P.O. Box 173, Al-Kharj, 11942 Saudi Arabia; 2 Institute of Research and Development, Duy Tan University, Da Nang, Vietnam; 3 School of Engineering and Technology, Duy Tan University, Da Nang, Vietnam

**Keywords:** Supercritical fluids, Predictive modeling, Data-driven solubility estimation, Bio-inspired optimizers, Hybrid regression systems, Green processing technologies, Chemistry, Engineering, Mathematics and computing

## Abstract

Reliable prediction of drug solubility in supercritical carbon dioxide (SC-CO_2_) is central to designing environmentally conscious pharmaceutical processes, yet experimental solubility measurements remain slow, resource-intensive, and often restricted to limited operating ranges. These constraints restrict the development of green technologies such as particle formation, controlled micronization, and solvent-free formulation strategies. This study introduces an automated computational framework that couples modern regression algorithms with bio-inspired optimization to deliver scalable solubility prediction across varying conditions. The approach employs Adaptive Boosting Regression and Light Gradient Boosting Regression as core learners, which are combined through hybrid ensemble schemes and tuned using two recent metaheuristic algorithms the Osprey Optimization Algorithm and the Artificial Protozoa Optimizer. Model behavior was assessed using repeated cross-validation, a suite of accuracy metrics (RMSE, R^2^, MDAPE, SI, NSE), non-parametric statistical comparison, and ANOVA-based sensitivity evaluation. A multi-criteria ranking using the TOPSIS method identified the APO-driven ensemble (ALAP) as the most reliable configuration, achieving RMSE = 0.191, R^2^ = 0.982, and MDAPE = 15.6% on the test set. Beyond its algorithmic design, the framework offers a practical computational alternative to extensive laboratory experimentation, providing a transferable, data-driven tool for efficient and eco-friendly pharmaceutical process design.

**Supplementary Information:**

The online version contains supplementary material available at https://doi.org/10.1038/s41598-026-59449-z.

## Introduction

The pharmaceutical market, such a technology like supercritical fluid $\:\left(SCF\right)$ offers an environmentally friendly and versatile processing platform. In reality, SCFs have remarkable physical properties such as tunable solvating power and diffusivity that is nearly that of liquids^1,2^. They are recognized as the ideal medium for enhancing solubility, extraction, and particle engineering due to their unique characteristics. Since of its moderate critical conditions ($\:Tc\:=\:304.1\:K,\:Pc\:=\:73.8\:bar$), non-toxicity, non-flammability, affordability, and ease of recovery, CO₂ SFC has been the most popular option among SFCs^3,4^. These characteristics have made it broadly relevant in a variety of fields, includ the separation of bioactive chemicals, food extraction, medication micronization, and nanoparticle manufacturing^5^.

In pharmaceutical research, the solubility of pharmaceuticals in $SC - CO_{2}$ plays a significant role in determining the viability of procedures like $\:RESS,\:SAS$, and * SSI*^6^. This is one of the prerequisites for the solubility data, which are typically regarded as essential for both improving the bioavailability and dissolution of medications with low water solubility and for developing new formulation routes that are both environmentally benign and scalable^7,8^. In the areas of narrower particle size distribution, absence of significant thermal stress, and absence of hazardous solvent residue, SC-CO₂-based micronization techniques usually outperform conventional ones (such as grinding, spray-drying, and freeze-drying)^9,10^. However, the degree to which the solubility of medications at various pressures and temperatures was understood greatly influenced the efficacy and authenticity of these techniques. Gravimetric, spectroscopic, or chromatographic investigations have historically been used to ascertain equilibrium solubility in SC-CO₂. Despite their accuracy, these tests are time-consuming, require a specifically designed high-pressure equipment, and are often limited to narrow process ranges. As a consequence, there is a dearth of experimental data, especially for complicated medicinal compounds, which makes it difficult to evaluate the findings and develop the procedure further^11^.

A comparative summary of the main research on drug solubility prediction in SC-CO₂ is shown in Table 1, which is divided into two categories: machine learning/AI-based techniques and experimental/thermodynamic approaches^12^. Equations of state (EoS) like Peng–Robinson and Soave–Redlich–Kwong, which accurately record solubility trends under various circumstances but often need exact calibration for particular compounds, have been the mainstay of experimental research^13,14^. On the other hand, machine learning techniques showed better generalization and prediction ability over larger datasets. The greatest accuracies were routinely attained by the Adaptive Neuro-Fuzzy Inference System (ANFIS)^15^, hybrid evolutionary deep learning models (e.g., GWO-DE-LSTM), and ensemble learning techniques (e.g., AdaBoost-GPR, bagging regressors), with R^2^ values regularly above 0.99^16,17^. The contrast shows how data-driven approaches have clearly replaced strictly thermodynamic models. Machine learning models, particularly ensemble and hybrid architectures, offer greater flexibility, accuracy, and applicability to a wider range of therapeutic molecules and scenarios, even as EoS and semi-empirical approaches remain significant due to their interpretability and physical foundation. This dual viewpoint emphasizes how physics-based and AI-driven methods work in tandem to improve accurate solubility prediction for medicinal applications.

**Table 1:** Comparative summary of experimental, thermodynamic, and machine learning approaches for predicting drug solubility in SC-CO₂.

Study	Drug(s)	Method/Data	Models/Techniques	Key findings
Experimental + Thermodynamic Models				
Sodeifian et al.^18^	Gemcitabine	Experimental (308–338 K, 120–270 bar)	Semi-empirical + EoS (Peng–Robinson, SRK)	Very low solubility; retrograde at 190–200 bar; PR best at low T, SRK at high T.
Laggoune et al.^19^	Sirolimus	Experimental (313–328 K, 12.5–25 MPa)	Semi-empirical, EoS (Sparks, SRK)	Direct solubility trend; Sparks & SRK best (Radj ≈ 0.978–0.980).
Sodeifian et al.^20^	5-FU	Experimental (308–338 K, 120–270 bar)	Semi-empirical, EoS, ML (DT, GB, RF, KRR)	Solubility ↑ with pressure; density-based model best (AARD = 4.12%).
Machine Learning and AI-Based Models				
Bahrami et al.^8^	Multiple solid drugs	1,816 data points (80–400 bar, 308–348.2 K)	GEP, ANFIS	ANFIS best (R^2^ ≈ 0.99, RMSE ≈ 0.26); MW most influential factor.
Alsaab & Althobaiti^21^	Phenytoin	Dataset: T, P, solubility, density	ARDr, GPR, LR + AdaBoost, JOA	ADA-GPR best (R^2^ = 0.996); ADA-LR (0.934), ADA-ARD (0.952).
Wu et al.^3^	Raloxifene	SC-CO₂ solubility & density	Bagging regressors: BRR, LR, PR + Tree-PEA	BAG-PR best (R^2^ = 0.986); others less precise.
Makarov et al.^5^	31,975 compounds (drug-like)	Large databank + thermo descriptors	CAT, Graph-based message-passing	Thermodynamic features improved accuracy and generalization.
Zhu et al.^22^	Busulfan	SC-CO₂ solubility	ANFIS (Trimf, Grid Partitioning)	ANFIS highly accurate for solubility prediction.
Najmi et al.^6^	Niflumic acid, Tolfenamic acid, Glibenclamide, Nystatin, Rivaroxaban	Experimental	PR, MLP, KNN + HS optimization	HS-PR best (R^2^ = 0.96449).
Alotaibi et al.^7^	Raloxifene	SC-CO₂ solubility & density	ET, RF, GB + gradient-based opt.	GB best for density (R^2^ = 0.986); ET best for solubility (R² = 0.949).
Damansabz et al.^23^	Compounds in SC-CO₂	T, P, density	XGBoost, DL, hybrid GWO-DE-LSTM	Hybrid model best (R^2^ = 0.931); T most important factor.
Bahrami et al.^8^	Solid drugs (general)	AI models	GEP, ANFIS	ANFIS best (R^2^ = 0.991 train, 0.990 validation); MW key factor.
Alsaab & Althobaiti^21^	Phenytoin	Dataset: T, P, solubility, density	LR, GPR, ARD + AdaBoost, JOA	ADA-GPR best (R^2^ = 0.996 solubility, 0.993 density).

**Fig. 1:**
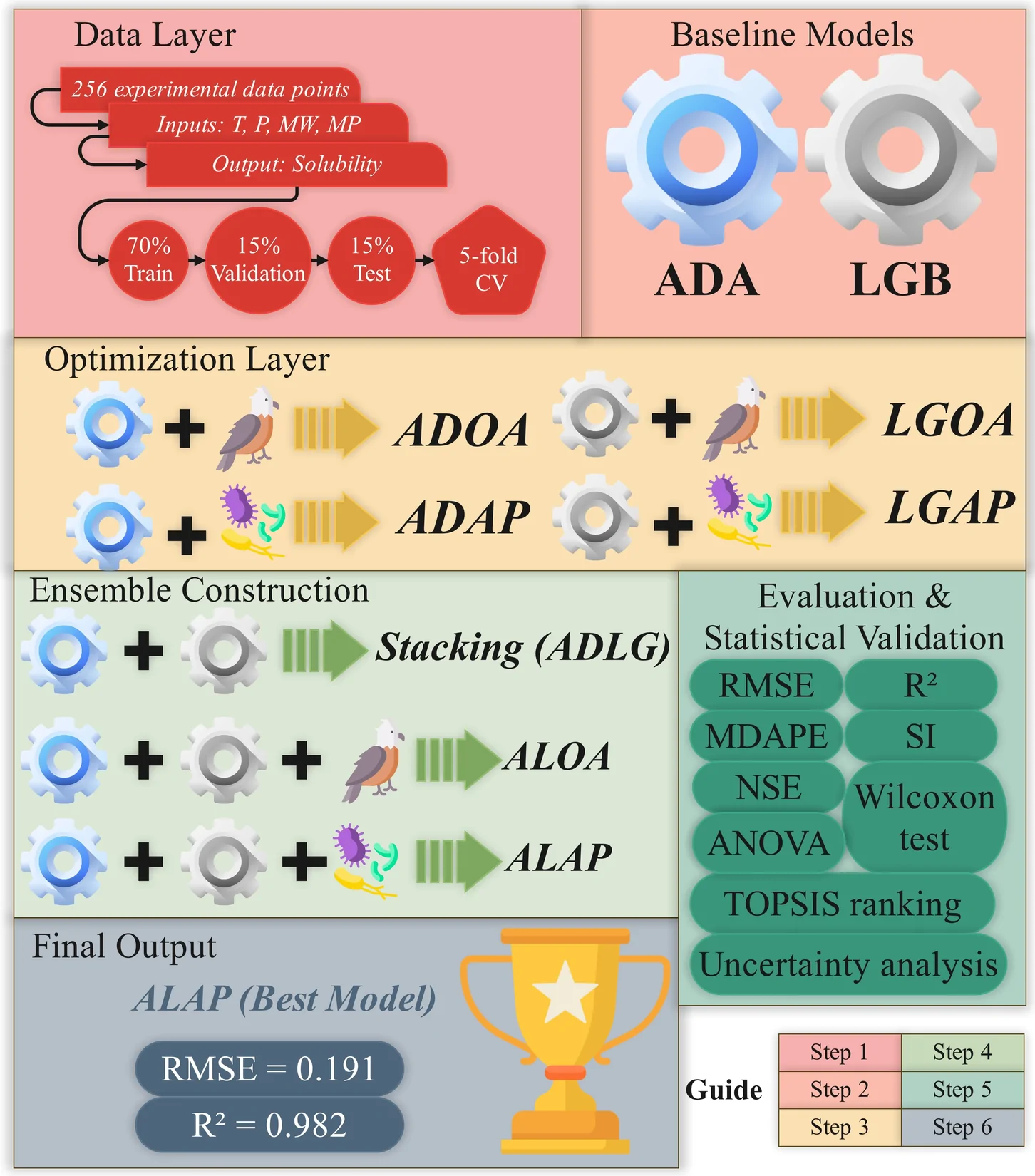
Overview structure of the study.

Previous studies on solubility prediction in supercritical carbon dioxide (SC-CO₂) have mainly focused on thermodynamic correlations, equations of state, and a range of machine learning models such as ANFIS, bagging-based regressors, and hybrid deep learning frameworks. While these approaches have achieved promising accuracy, they often lack robustness, comprehensive validation, and statistical reliability across diverse conditions. Moreover, although several existing studies have employed optimization techniques and statistical error metrics, the application of recently developed bio-inspired metaheuristics for hyperparameter tuning remains limited in this field. In addition, while sensitivity analysis or uncertainty assessment has been reported in some works, few studies have combined multi-criteria decision-making, nonparametric hypothesis testing, and sensitivity analysis within a unified validation framework for solubility prediction. To address these gaps, the objective of this study is to develop optimization-driven ensemble models that combine boosting-based learners with newly introduced metaheuristic algorithms to achieve highly accurate and generalizable solubility predictions. The novelty of this work lies in employing the Osprey Optimization Algorithm (OOA) and Artificial Protozoa Optimizer (APO) for the first time in SC-CO₂ solubility prediction, designing a hybrid ensemble model that significantly outperforms both single learners and conventional stacking approaches, and incorporating multi-criteria evaluation techniques such as TOPSIS, Wilcoxon test, and ANOVA sensitivity analysis to ensure statistical rigor. This integrated framework not only advances the methodological landscape of solubility modelling but also establishes a generalizable approach that can be extended to other physicochemical property predictions in pharmaceutical science. Figure 1 presents overview structure of the study.

## Materials and methods

### Data description

The dataset compiled for this study consists of 256 experimental solubility measurements for seven pharmacologically relevant drugs in SC-CO₂ (Table 2). These drugs, Famotidine (FAM), Erlotinib (ERH), Clonazepam (CLP), Nystatin, Gemifloxacin, Favipiravir (FVP), and Montelukast, represent diverse chemical structures and therapeutic classes, ensuring heterogeneity in the modelling inputs and outputs. The number of samples per drug ranges from 28 to 57, with solubility values obtained under varying conditions of temperature, pressure, and co-solvent composition. Experimental procedures followed standardized high-pressure equilibrium cell methodologies, with solubility quantified spectrophotometrically using drug-specific calibration curves. All measurements were performed in triplicate to minimize experimental uncertainty, thereby enhancing data reliability. For model development, the dataset was randomly divided into three subsets following a 70–15–15 ratio, corresponding to training, validation, and testing partitions. The training set (179 samples) was used to fit the machine learning models, the validation set (38 samples) guided hyperparameter tuning and model optimization, and the independent test set (35samples) enabled an unbiased evaluation of predictive performance. This partitioning ensured both sufficient data availability for training and reliable assessment of model generalization.

**Table 2:** Overview of drugs, sample sizes, chemical formulas, and solubility experimental procedures.

Ref	Drug	No. of samples	Formula	Summary of experimental procedure
^24^	Famotidine (FAM)	28	C_8_H_15_N_7_O_2_S_3_	Solubility measured in a high-pressure batch flow system. 2000 mg drug loaded into 300 mL cell; CO_2_ liquefied, pressurized, and equilibrated (135 min, 100 rpm stirring). Saturated solution withdrawn via loop, depressurized into methanol (5 mL). Solubility analyzed at λmax 265 nm (triplicate runs)
^25^	Erlotinib (ERH)	28	C_22_H_23_N_3_O_4_	3000 mg drug mixed with SC-CO_2_ in 300 mL cell. CO₂ cooled, liquefied, compressed, and equilibrated (120 min). Saturated SC-CO₂ sampled (600 µL) into methanol (5 mL). Loop rinsed with solvent. Solubility determined spectrophotometrically at λmax using calibration curves (triplicates)
^26^	Clonazepam (CLP)	28	C_15_H_10_ClN_3_O_3_	3000 mg drug mixed with SC-CO_2_ in 300 mL equilibrium cell. CO_2_ filtered, liquefied, and pressurized to 30 MPa. Equilibrated for 130 min at controlled T (308–338 K). Saturated CO_2_ sampled (500 µL) into methanol (5 mL). Solubility measured by UV–Vis at λmax using calibration curve (triplicates)
^27^	Nystatin	28	C_47_H_75_NO_17_	3000 mg drug with ethanol co-solvent (3 mol%) in 300 mL cell. CO_2_ liquefied, pressurized, and introduced. Equilibrated for 90 min with stirring. Saturated SC-CO_2_ sampled (500 µL) into methanol (5 mL). Solubility measured at 360 nm with calibration (triplicates)
^28^	Gemifloxacin	28	C_18_H_20_FN_5_O_4_	~ 1 g drug mixed with SC-CO₂ in equilibrium cell. CO₂ filtered, liquefied, pressurized (120–300 bar), equilibrated (160 min, 308–338 K). Saturated solution depressurized into methanol. Solubility determined by UV–Vis at λmax 269 nm
^29^	Favipiravir (FVP)	56	C_5_H_4_FN_3_O_2_	3000 mg drug with ethanol (1–3 mol%) in a 300 mL cell. CO_2_ liquefied, filtered, pressurized (up to 40 MPa). Equilibrated for 90 min with stirring. Saturated SC-CO_2_ sampled into ethanol/methanol (5 mL). Solubility measured spectrophotometrically using calibration curves (triplicates)
^29^	Montelukast	56	C_35_H_36_ClNO_3_S	Same setup as Favipiravir: 3000 mg drug with co-solvent in a 300 mL high-pressure equilibrium cell. CO_2_ liquefied, compressed, equilibrated (90 min). Saturated SC-CO_2_ was depressurized into a methanol solution (5 mL). UV–Vis spectrophotometry is used for solubility determination

By integrating data from multiple sources and experimental designs, the dataset provides a robust foundation for developing optimization-driven ensemble models capable of capturing the nonlinear solubility behaviour of structurally diverse drugs in SC-CO_2_. The selection of the seven pharmaceuticals was based on three main criteria: data availability, physicochemical diversity, and pharmaceutical relevance. Only compounds for which reliable experimental solubility data in SC-CO₂ were reported using comparable high-pressure equilibrium methods were included. Furthermore, the selected drugs span a broad range of molecular weights, melting points, and chemical functionalities, including heterocyclic, macrolide, fluorinated, and lipophilic structures. This diversity ensures adequate heterogeneity in the input space and enhances the generalizability of the developed predictive models. In addition, the chosen compounds belong to different therapeutic classes and are relevant to supercritical processing applications such as micronization and particle engineering.

**Fig. 2:**
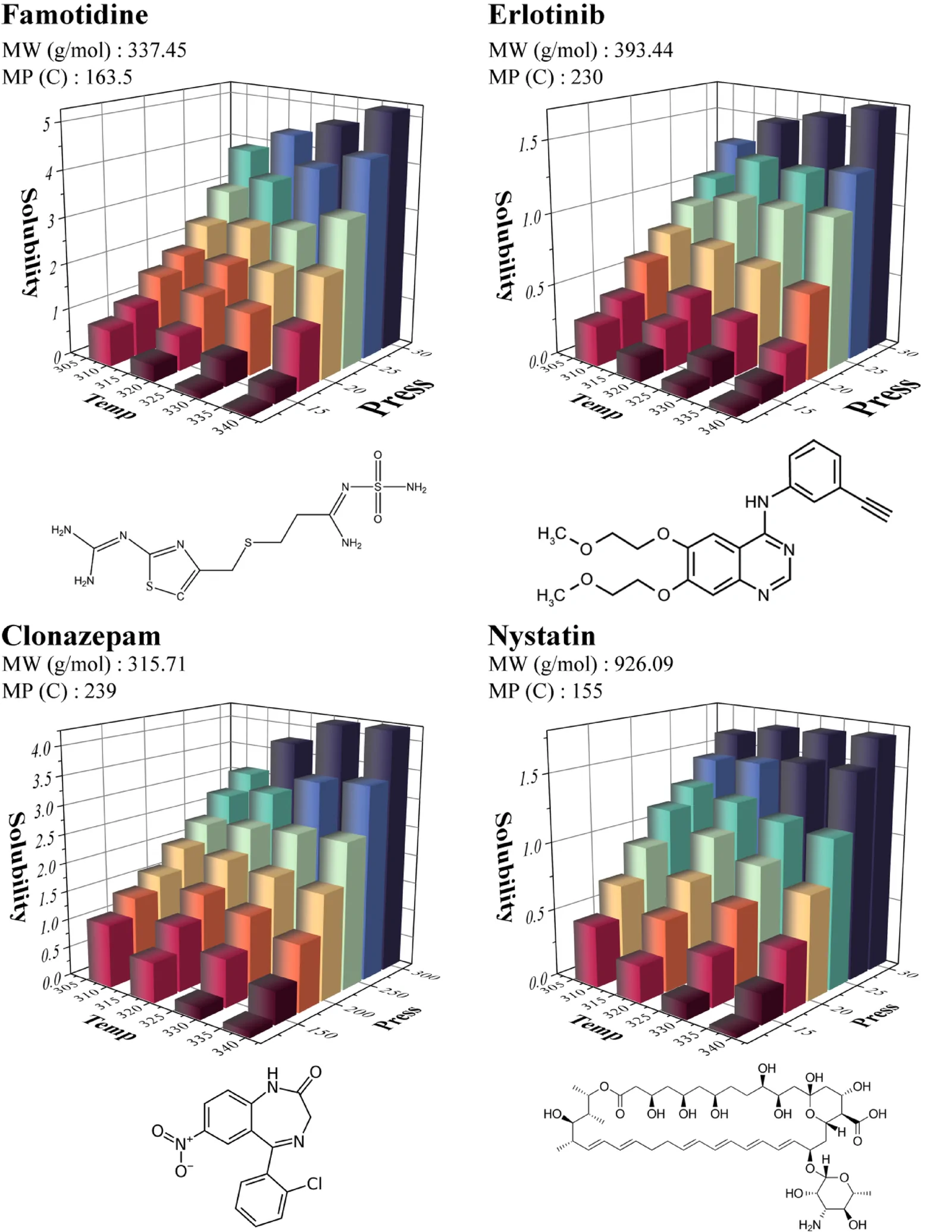

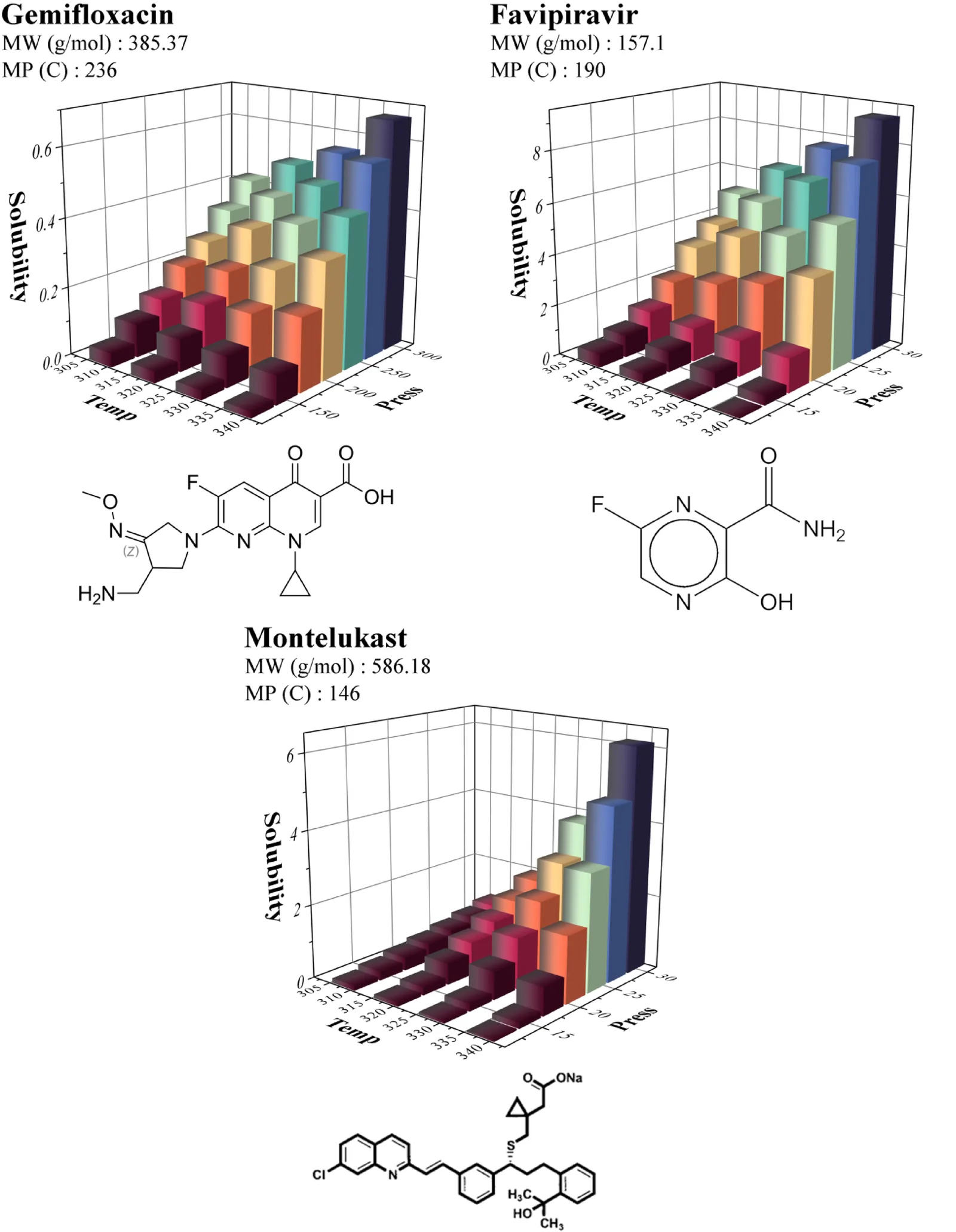
Solubility behaviour of selected drugs as a function of temperature and pressure. Each subplot illustrates the three-dimensional solubility surface alongside the molecular structure, molecular weight (MW), and melting point (MP) of the compound.

Figure 2 provides a clear visualization of how solubility varies with temperature and pressure, two critical thermodynamic parameters influencing drug behaviour in SC-CO_2_. In all cases, solubility tends to increase with rising pressure, which is consistent with the enhanced density and solvent power of SC-CO₂ at higher pressures. The influence of temperature is more compound-specific, reflecting the interplay between drug sublimation/vaporization tendencies and the density reduction of SC-CO₂ at elevated temperatures. Drugs with lower MW and MP generally display higher solubility, while those with bulky structures and high polarity exhibit lower solubility, highlighting the role of molecular characteristics in governing solubility outcomes.

For instance, the solubility surface for Clonazepam demonstrates a strong positive dependence on both temperature and pressure. At higher pressures, solubility rises sharply, indicating that density-driven solvent power dominates the dissolution process. The effect of temperature is also significant, as higher thermal energy enhances the sublimation tendency of Clonazepam, facilitating its transition into the SC-CO₂ phase. Its relatively moderate molecular weight and aromatic heterocyclic structure contribute to a higher solubility range compared to larger biomolecules. In contrast, Nystatin shows a much lower solubility scale, with values remaining under 2 across all conditions. Despite having a lower melting point, its very high molecular weight and large polyene–polyketide structure significantly reduce its solubility in SC-CO₂. The increase in solubility with pressure remains evident, but the effect of temperature appears less pronounced compared to Clonazepam, likely due to the limited volatility of this bulky macrolide compound. These results highlight the difficulty of dissolving large bioactive molecules in SC-CO₂ without the use of co-solvents or modifications.

### K-fold cross-validation

Table 3 presents the outcomes of 5-fold cross-validation for the baseline models (ADA and LGB). The R² values across different folds indicate that both models achieved high predictive capability, with average R² scores above 0.93 in all cases. ADA consistently outperformed LGB across the folds, achieving peak performance at K3 (R² = 0.959) and maintaining stable results throughout (range: 0.940–0.959). LGB also demonstrated reliable performance, with R² ranging from 0.932 to 0.944, though with slightly higher variability compared to ADA. The fold-wise averages reveal that ADA exhibits both stronger predictive accuracy and better robustness than LGB, as evidenced by higher mean R² values in four out of five folds. Importantly, the narrow range of R² fluctuations across folds suggests that both models generalize well to unseen data, minimizing the risk of overfitting. These findings reinforce the suitability of boosting-based regressors as strong baseline learners, providing a solid foundation for their integration into more advanced stacking and hybrid ensemble frameworks.

**Table 3:** The result of the developed K-Fold.

Indicator	Models	Number of K-Folds				
		*K1*	*K2*	*K3*	*K4*	*K5*
R^2^	ADA	0.945	0.955	**0.959**	0.946	0.940
	LGB	0.941	**0.944**	0.939	0.932	0.941
	Average	0.943	**0.950**	0.949	0.939	0.941

The highest R^2^ value among the folds are in bold.

### Light Gradient Boosting (LGB)

LGB is a relatively recent gradient boosting framework that has gained prominence due to its efficiency, scalability, and predictive accuracy^30,31^. Built from decision tree learners that are combined by boosting methods, LGB continuously reduces error by paying attention to incorrectly classified or poorly predictive instances, accumulating over time a strong predictive model from a collection of weak learners. Its primary innovations are memory optimization, speed of calculation, and improved handling of complex, nonlinear data relationships. One of the most significant contributions of LGB is its histogram-based construction of decision trees. Instead of relying on continuous feature values, the algorithm transforms them into bins that significantly reduce memory consumption and computing costs. The binning not only accelerates training but also provides regularization by smoothening feature borders such that overfitting is suppressed^32^. Contrary to previously suggested boosting methods, LGB abolishes the need for pre-sorted features such that an effective parallelization for large-scale deployment is allowed.

Its leaf-like development technique is another characteristic. In contrast to traditional algorithms that enlarge decision trees by levels, LGB splits a decision tree after determining which leaf receives the most information benefit. With fewer iterations, this method creates deeper and narrower trees that allow the model to detect subtle feature interactions. Despite this, deeper trees will increase the danger of overfitting; to achieve this, LGB adds extra regularization parameters and explicit depth limitations that strike a compromise between the flexibility and generalizability of trees. Because of these characteristics, LGB is especially well-suited for high-dimensional, heterogeneous datasets, as those seen in solubility modeling and pharmaceutical informatics. Its extensive use in sophisticated ensemble and hybrid constructions is made possible by the efficient processing of molecular descriptors combined with predictive resilience. LGB served as a baseline learner in the task at hand and was also used later on in blending optimization-driven ensembles, confirming its applicability for capturing nonlinear solubility behavior in structurally varied medicinal molecules.

### Adaptive Boosting Regression (ADAR)

ADAR, first introduced by Freund and Schapire^33^, is one of the most influential ensemble learning techniques for improve the predictive performance of weak learners. The essential concept of AdaBoost lies in its iterative reweighting approach such that asequence of weak regressors is sequentially learned, each focusing greater attention on instances that were misclassified by its antecedents. Through iteratively varying sample weights across iterations, the algorithm concentrates learning capacity on hard-to-predict observations, concluding with a strong composite learner from separately simple learners. In the regression case, the ultimate model is constructed by an ADAR as a weighted sum of its learner components such that each learner contributes proportionate to its predictive strength. Although each separate weak learner minimally outperforms random guessing, their collective contribution yields a very accurate and robust regressor. This property of an ADAR makes it extremely effective for datasets with intricate, nonlinear relationships wherein individual learners tend to underperform. One of the most popular choices of the learner for an ADAR is the decision tree regressor due to its simplicity, explainability, as well as its capability of modeling nonlinear interactions.

In the current work, decision trees were employed as the foundation of the ADAR framework. Their union with the boosting mechanism allowed the model iteratively to refine remaining errors such that it could achieve higher predictive accuracy than each of them individually could offer.The strength of ADAR lies not only in its accuracy but also its flexibility. Through its real-time redistribution of emphasis towards more stressful cases, the algorithm ensures balanced learning regardless of varying data distributions. This property is best exploited in pharmaceutical solubility prediction, where datasets tend to be heterogenous across molecular structure as well across thermodynamic condition. In the current framework, ADAR served as a baseline learner and was shown to demonstrate steady predictive potential such that a stable baseline against which hybrid models as well as optimization-guided ensemble models could be constructed could be obtained.

### Artificial Protozoa Optimizer (APO)

The * APO* is a recently developed bio-inspired metaheuristic algorithm that mimics the adaptive survival strategies of protozoa, particularly their ability to alternate between different nutritional and reproductive modes in response to environmental conditions^34^. Drawing inspiration from the life cycle of Euglena, * APO* framework integrates four primary behavioral mechanisms foraging, autotrophy, heterotrophy, dormancy, and reproduction into a unified optimization strategy. In the foraging phase, protozoa regulate their environment by balancing internal forces with external pressures such as competition or limited resources. The phase allows candidate ‏solutions to spread out while holding off converging too soon. The autotrophic mode emulates the ability of protozoa to generate energy by photosynthesis when light favors it. In optimization terms, that means self-directed movements towards favorable parts of the search space driven by solution quality ‏and environment ‏feedbacks. The heterotrophic mode conversely emulates energy acquisition by consumption of external ‏organic matter by mimicking exploitation of information from nearby ‏solutions for enhanced search ‏precisiveness.

Dormancy mechanism enables protozoa to withstand unfavorable circumstances by entering a dormant state, which for APO is simulated by reinitializing poor-quality solutions within given bounds to secure population diversity. Last but not least, the reproduction process that imitates binary fission enables successful protozoa to duplicate and integrate controlled perturbations so that discovery of unexplored areas comes after exploitation of promising opportunities. An adaptive balance between exploration and exploitation is provided by these behavioral patterns. APO maintains solution variety while steadily moving closer to global optima via the dynamic switching between feeding strategies and survival mechanisms. The approach is very suited for complicated, nonlinear optimization problems, such as those seen in tasks like hyperparameter tweaking of high-end machine learning models, because to its population stability and flexibility. APO was initially used in this study to solve the drug solubility prediction issue, and it outperformed conventional optimizers in terms of resilience and convergence dynamics.

### Osprey Optimization Algorithm (OOA)

OOA is a bio-inspired metaheuristic that was derived from the characteristic fishing behaviors of ospreys, a fish-specialized bird of prey. Ospreys possess remarkable vision and agility that enable them to notice fish below the water surface, dive at high speed, then carry their prey to safety at feeding areas. These behaviors sharp piercing detection, selective capture, and safe transport were codified into algorithmic guidelines and employed to create OOA as an intelligent optimizer that could balance explorations of the landscape globally while exploitation locally^35^. OOA is a population-based algorithm, where each candidate solution is represented as an “osprey” positioned within the search space^36^. The optimization process begins with random initialization, ensuring diversity across the population. Each solution is evaluated using the objective function, and the quality of these solutions determines their likelihood of being updated or replaced during subsequent iterations.

The algorithm proceeds in two primary phases. The first phase, exploration, simulates the osprey’s process of scanning for fish and executing a dive. Here, solutions are guided toward promising regions of the search space, while stochastic perturbations prevent premature convergence to local optima^37^. This phase ensures broad coverage of the problem domain, analogous to the osprey’s aerial survey of its aquatic environment. The second phase, exploitation, reflects the osprey’s behavior of transporting its captured fish to a safe location for consumption. In computational terms, this corresponds to refining candidate solutions around the most promising regions identified during exploration. This mechanism enhances convergence stability, allowing OOA to progressively focus search efforts on near-optimal solutions.

By alternating between these two phases, OOA achieves a good trade-off between diversification and intensification. Owing to its biologically inspired construction, OOA is particularly effective for high-dimensional and nonlinear optimisation problems. OOA was employed as an optimiser for fine-tuning instances of machine learning models in this work, and it contributed towards improved convergence efficiency as well as predictive validity for drug solubility modelling.

### Stacking ensemble

Stacking, also referred to as stacked generalization, is an ensemble learning paradigm that integrates multiple base learners into one predictive model. Unlike simpler ensemble techniques such as bagging or boosting, which combine learners by means of averaging or sequential reweighting, stacking involves the use of a hierarchical method by which the results of multiple base models’ predictions are chief inputs of a higher-level learner referred to as the meta-learner. Such an organization enables the model to discern complementary strengths of different algorithms, thus reducing bias and variance while improving overall generalizability. The procedure starts with the separate training of multiple-base learners of different structure over the same data. As long as they provide diverse patterns of prediction, the learners may have different structures, such as decision-tree-based regression, boosting techniques, or kernel approaches. The outcomes of these basic models are then combined to create a fresh data set that serves as the meta-learner’s input. In order to address their individual shortcomings and balance their input towards contextual relevance, the meta-learner receives training after discovering systematic correlations between the base predictions. Combining LGB and ADAR as base learners with a regression-based meta-learner that combined their outputs was known as stacking. This setup was selected to avoid overfitting risks by using weighted summing and to take use of the efficient error-reduction capabilities of boosting techniques. The stacked structure was additionally shielded against information leakage and overly optimistic assessment by using k-fold cross-validation during training. The flexibility to combine models with different error profiles is the main advantage of stacking for drug solubility prediction. Although individual learners are prone to poor performance on individual compound subsets or thermodynamic condition sets, the stacked ensemble is able to counter such imbalances by enhancing both robustness and accuracy. As demonstrated for the analyses that followed, the ensemble of stacking scored a remarkable performance gain over single learners, offering a robust baseline at an intermediate stage prior to construction of hybrid optimization driven ensembles.

### Evaluation metrics

To ensure a rigorous and balanced assessment of model performance, five complementary statistical indicators were employed: Root Mean Square Error $\:\left(RMSE\right)$, Coefficient of Determination $\left( {R^{2} } \right)$, Median Absolute Percentage Error $\:\left(MDAPE\right)$, Scatter Index $\:\left(SI\right),$ and Nash–Sutcliffe Efficiency $\:\left(NSE\right)$. These metrics collectively capture error magnitude, explanatory power, scale normalization, and predictive reliability. Their descriptions and mathematical formulations are summarized in Table 4. By integrating these indicators, the evaluation framework ensures balanced judgment across error sensitivity, scale independence, and explanatory adequacy. The mathematical definitions of the metrics are provided below, where $\:{y}_{i}$ represents the observed values, $\:\widehat{{y}_{i}}$ the predicted values, $\:\stackrel{-}{y}$ the mean of observed values, and * n* the total number of samples:

**Table 4:** Evaluation metrics used in this study.

Metric	Description	Formula
RMSE (Root Mean Square Error)	Measures the average magnitude of prediction errors, sensitive to large deviations.	$\:RMSE=\sqrt{\frac{1}{n}{\sum\:}_{i=1}^{n}{({y}_{i}-\widehat{{y}_{i}})}^{2}}$
R^2^ (Coefficient of Determination)	Indicates the proportion of variance in observed values explained by the model.	$\:{R}^{2}=1-\frac{{\sum\:}_{i=1}^{n}{({y}_{i}-\widehat{{y}_{i}})}^{2}}{{\sum\:}_{i=1}^{n}{({y}_{i}-\stackrel{-}{y})}^{2}}$
MDAPE (Median Absolute Percentage Error)	Provides robust relative error estimation, less sensitive to outliers than * MAPE*.	$\:MDAPE=median\left(\left|\frac{{y}_{i}-\widehat{{y}_{i}}}{{y}_{i}}\right|\times\:100\right)$
SI (Scatter Index)	Normalizes * RMSE* by the mean of observed values for scale-independent evaluation.	$\:SI=\frac{RMSE}{\stackrel{-}{y}}$
NSE (Nash–Sutcliffe Efficiency)	Compares predictive performance against the mean of observations; closer to * 1* indicates higher reliability.	$\:NSE=1-\frac{{\sum\:}_{i=1}^{n}{({y}_{i}-\widehat{{y}_{i}})}^{2}}{{\sum\:}_{i=1}^{n}{\left({y}_{i}\right)}^{2}}$

### Justification of methods and optimizations

Here strategy employed was motivated by the scientific necessity of solvent solubility ‏prediction in supercritical CO₂ as much as by weaknesses there expressed by earlier works. The choices weren’t arbitrary but instead a compromise of hard theory with operational reliability for pharmaceutical application. Boost algorithms notably ADARand LGB were selected for a place to begin since they’ve been good at discerning complex nonlinear data patterns.Each brings a complementary strength: ADAR systematically shifts attention to difficult-to-predict cases, while LGBR offers efficient handling of high-dimensional descriptors through leaf-wise tree growth. Together, they provided a strong foundation for further refinement.

However, one drawback of these algorithms is their reliance on traditional parameter-tuning techniques, which often fall short of identifying the best model parameters. The OOA and the APO are two new bio-inspired optimizers that were used to get around this. While APO mirrors protozoa’s adaptive survival techniques to preserve variety and prevent premature convergence, OOA represents the osprey’s capacity to strike a compromise between pinpoint catch and wide exploration. The research obtained higher convergence and predictive stability by including these optimizers into our framework, which also led to more dependable hyperparameter tuning.

The hyperparameter tuning of the hybrid ensemble models was formulated as a single-objective optimization problem. During the optimization process, the Artificial Protozoa Optimizer (APO) minimizes the prediction error of the model using the Root Mean Square Error (RMSE) as the objective function, evaluated on the validation dataset. The objective function is defined as:

1 $$\:\underset{\boldsymbol{\theta\:}}{\mathrm{m}\mathrm{i}\mathrm{n}}{\hspace{0.25em}\hspace{0.05em}}f\left(\boldsymbol{\theta\:}\right)=\sqrt{\frac{1}{N}\sum\:_{i=1}^{N}{\left({y}_{i}-{\widehat{y}}_{i}\left(\boldsymbol{\theta\:}\right)\right)}^{2}}$$

where $\:{\mathrm{y}}_{\mathrm{i}}$denotes the experimental solubility, $\:{\widehat{\mathrm{y}}}_{\mathrm{i}}\left({\uptheta\:}\right)$represents the predicted solubility obtained from the ALAP model with hyperparameter set $\:{\uptheta\:}$, and $\:\mathrm{N}$is the number of validation samples. Although multiple statistical indicators (R², MDAPE, SI, and NSE) are used to evaluate and rank the models, they are not combined into the optimization objective and therefore do not constitute a multi-objective optimization problem.

From here on, stacking also appeared as an interim step towards combining ADAR and LGBR. Although stacking provided useful gains by combining error patterns, here took a step further by building hybrid ensembles (ALOA and ALAP) such that optimization and aggregation were integrated. This step proved essential: rather than only averaging or weighing predictions, the hybrids allowed each learner to operate in an optimized space such that steady improvements were earned across train, validation, and test. Finally, evaluation relied upon something more than trivial error measures. Sensitivity analysis (ANOVA), nonparametric statistical testing (Wilcoxon signed-rank test), and multi-criteria decision-making (TOPSIS) were all used to ensure that the models were not only accurate but also statistically sound. These levels of validation helped to guarantee that the benefits this research documented were significant, repeatable, and broadly applicable.

### Uncertainty estimation

Because minor changes may hinder formulation and process design, it is necessary to explicitly include uncertainty when predicting the solubility of pharmaceuticals in supercritical CO₂. Experimental variance, compound heterogeneity, algorithm bias, and susceptibility to hyperparameter adjustment are some of the sources of uncertainty. They were included using error distribution statistics to measure agreement with experimental data and k-fold cross-validation to forecast sampling variance. ANOVA-based sensitivity analysis predicted the contribution of both molecular and thermodynamic descriptors, whereas the Wilcoxon signed-rank test indicated whether measured and expected values differed substantially. Additionally, TOPSIS ranking included a variety of performance indicators to provide rigorous and equitable model comparison. A thorough understanding of prediction dependability was offered by this multi-layered method. The approach verified that the APO-driven hybrid ensemble coupled accuracy and resilience, providing a reliable tool for solubility prediction in pharmaceutical research, by clearly defining sources of variability.

### Software implementation and computational cost

All simulations and model implementations were carried out using the Python programming language on a Windows 11 operating system. The computational platform consisted of an Intel Core i7 processor, 16 GB RAM, a 1 TB solid-state drive (SSD), and an NVIDIA RTX 3050 GPU with 6 GB memory. The machine learning models and metaheuristic optimizers were implemented using standard Python scientific computing libraries. The computational cost varies according to model complexity. Training the baseline ADA and LGB models requires the shortest runtime, typically on the order of seconds. The simple ensemble model (ADA + LGB) incurs a moderate increase in computation time due to the combination of two learners. Hybrid models optimized using the Osprey Optimization Algorithm (OOA) and Artificial Protozoa Optimizer (APO) require additional time because of their iterative population-based search procedures. The fully optimized hybrid ensemble (ALAP), which simultaneously tunes base learners and ensemble weights, exhibits the highest computational cost, typically on the order of several minutes per training run. Despite this increase, the computational demand remains acceptable for offline pharmaceutical solubility modeling and is compensated by the substantial improvement in predictive accuracy and stability achieved by the optimized hybrid ensemble.

## Results and discussion

The results highlight the predictive capability of the developed machine learning frameworks, combining single models, hybrid variants, and ensemble approaches. Two baseline learners, ADA and LGB, were initially constructed as reference models. To enhance their performance, hybrid versions were developed using advanced metaheuristic optimizers. Furthermore, stacking and ensemble frameworks were implemented, including ADLG (ADA + LGB), ALOA (ADA + LGB+OOA), and ALAP (ADA + LGB+APO), which aimed to leverage complementary strengths of different learners and optimizers. Model evaluation was performed across training, validation, and testing phases using statistical indicators.

**Fig. 3:**
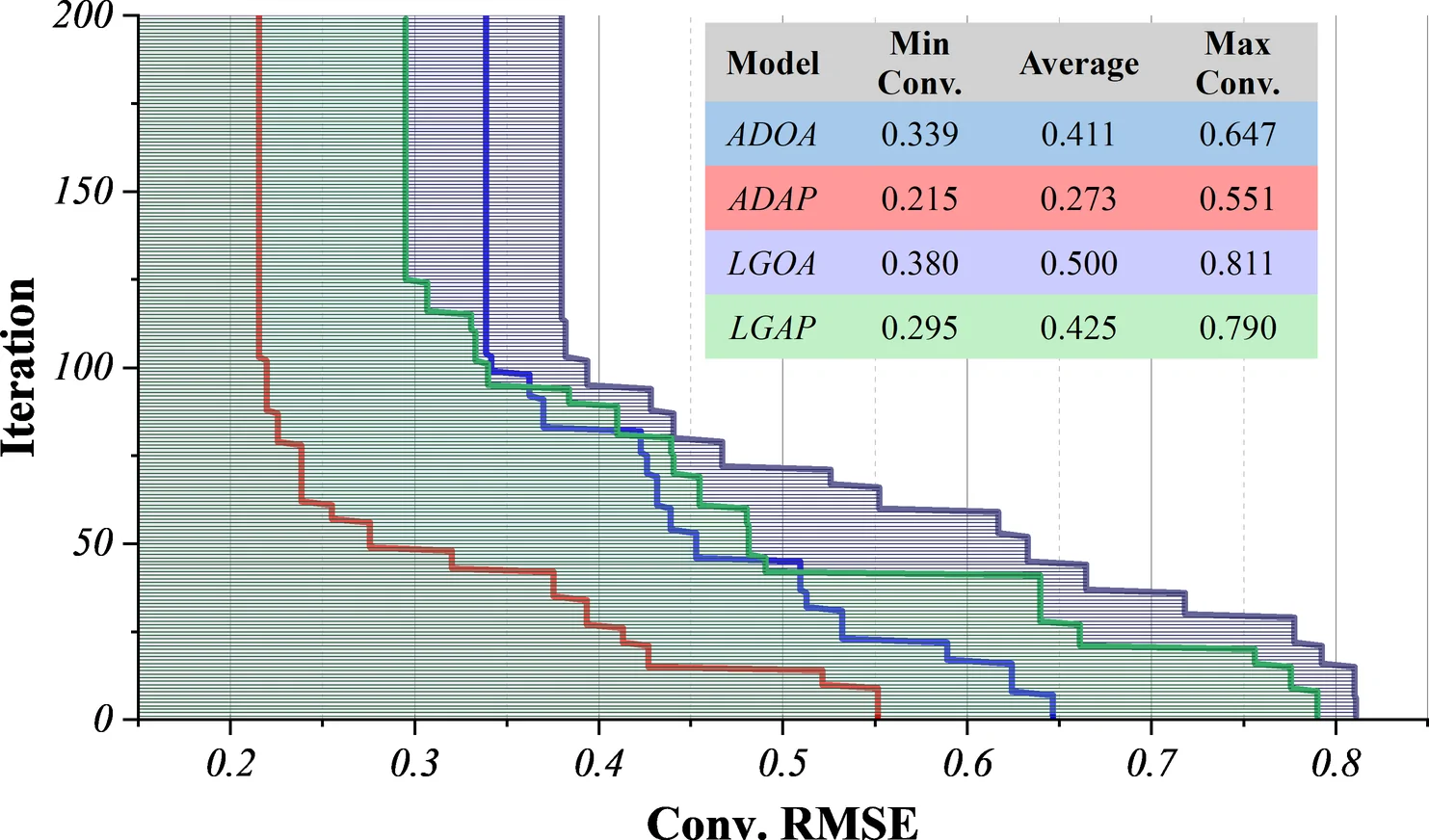
Line for the convergence behaviour of the models during optimization.

Figure 3 illustrates the convergence behaviour of the hybrid models during optimization, while Table 5 lists the final hyperparameter configurations obtained through metaheuristic search. The convergence trajectories reveal that all models progressively reduced their RMSE values within 200 iterations, though with notable differences in efficiency and stability. Among them, ADAP achieved the most rapid and stable convergence, reaching the lowest minimum RMSE (0.215) with a narrow fluctuation band (0.215–0.551). This indicates that the APO-driven ADA ensemble not only attained superior accuracy but also exhibited more consistent optimization dynamics. In contrast, LGOA required more iterations and exhibited wider RMSE variability (0.380–0.811), indicating slower convergence and less stable performance, although it ultimately achieved competitive accuracy. The hyperparameter profiles in Table 5 complement these findings. ADAP adopted a relatively high number of estimators (279) and a moderate learning rate (0.24), enabling effective boosting while avoiding overfitting. Similarly, LGAP benefited from a larger number of leaves (492) and estimators (679), along with a finely tuned learning rate (0.466), which contributed to improved convergence compared to LGOA. Interestingly, the extremely high max bin values assigned to the LGB-based models (up to 694,000) highlight the optimizer’s preference for finer feature discretization to capture subtle descriptor interactions.

**Table 5:** Hyperparameters of the hybrid models with their assigned values.

Hybrid models	Hyperparameter					
	N estimators			Learning rate		
ADOA	126			0.28		
ADAP	279			0.24		

	Num leaves	Max depth	Learning rate		N estimators	Max bin
LGOA	213	148	0.361		125	694,000
LGAP	492	145	0.46621		679	571,000

Table 6 summarizes the performance of all models across training, validation, and test phases, evaluated using RMSE, R², MDAPE, SI, and NSE. The results clearly demonstrate the progressive improvement achieved by moving from single learners to hybrids and then to hybrid ensembles. Single models (ADA and LGB) provided solid baselines, achieving R² values ranging from 0.922 to 0.958. However, their relatively higher RMSE (0.334–0.455) and larger MDAPE (20–39%) highlight limitations in capturing complex nonlinear relationships. Hybrid models (ADOA, ADAP, LGOA, LGAP) exhibited substantial performance gains, particularly ADAP and LGAP. For example, ADAP improved the R² to 0.974 on validation and reduced the RMSE to 0.220, while LGAP achieved the lowest MDAPE in both training (13.83%) and validation (14.60%). These results underscore the benefit of metaheuristic-driven parameter tuning, which enabled the models to extract more informative patterns from molecular descriptors. Stacking (ADLG) further enhanced predictive accuracy compared to single models; however, its performance remained inferior to that of metaheuristic-optimized hybrids.

This indicates that linear aggregation of learners alone is insufficient without advanced parameter optimization. Hybrid ensembles delivered the most notable improvements. The OOA-driven ensemble (ALOA) consistently outperformed stacking, achieving an R² of 0.977 and an RMSE of 0.282 on the test set. The APO-driven ensemble (ALAP), however, emerged as the top-performing model across all phases, with exceptional consistency. On the test set, ALAP achieved RMSE = 0.191, R² = 0.982, MDAPE = 15.61%, SI = 0.153, and NSE = 0.979, reflecting both accuracy and robustness. Importantly, ALAP maintained stable performance across training and validation, avoiding signs of overfitting while delivering superior generalization to unseen compounds. Taken together, these results confirm that integrating boosting-based learners with APO optimization provides a powerful predictive framework. The consistent superiority of ALAP across all statistical indicators highlights its reliability and suitability for practical solubility prediction tasks, advancing beyond conventional ML and ensemble strategies.

To quantify the contribution of the OOA and APO, the performance of baseline models was compared with that of their optimized counterparts. The non-optimized ADA and LGB models yielded RMSE values of 0.334 and 0.382 on the test set, respectively. After optimization, ADOA and LGOA reduced these errors to 0.305 and 0.345, while APO-based models (ADAP and LGAP) further decreased RMSE to 0.233 and 0.301, respectively. The hybrid ensemble optimized by APO (ALAP) achieved the lowest RMSE of 0.191 and the highest R² of 0.982. These results demonstrate that metaheuristic optimization substantially enhances predictive accuracy beyond conventional model training. In particular, APO provided a more consistent improvement than OOA, suggesting a stronger balance between exploration and exploitation in the hyperparameter search process. Therefore, the benefit of the proposed optimizers is not conceptual but quantitatively reflected in the improved prediction accuracy and stability of the optimized models.

**Table 6:** Performance metrics of the models, assessing their predictive accuracy and effectiveness using key statistical indicators.

Process	Category	Models	Evaluation metrics				
			RMSE	R^2^	MDAPE	SI	NSE
Train	Single models	ADA	0.402	0.958	31.419	0.221	0.958
		LGB	0.455	0.947	21.883	0.250	0.946
	Hybrid models	ADOA	0.349	0.974	22.350	0.192	0.968
		ADAP	0.213	0.988	14.897	0.117	0.988
		LGOA	0.390	0.962	16.857	0.214	0.960
		LGAP	0.296	0.978	13.830	0.163	0.977
	Stacking *(ADA + LGB)*	ADLG	0.365	0.967	15.569	0.201	0.965
	Hybrid ensemble *ADA + LGB+OOA*	ALOA	0.333	0.975	15.842	0.183	0.971
	Hybrid Ensemble *ADA + LGB+APO*	ALAP	0.202	0.990	9.918	0.111	0.989
Validation	Single models	ADA	0.392	0.926	39.490	0.292	0.913
		LGB	0.391	0.914	20.394	0.291	0.914
	Hybrid models	ADOA	0.279	0.959	20.684	0.208	0.956
		ADAP	0.220	0.974	25.548	0.164	0.973
		LGOA	0.328	0.942	19.072	0.244	0.939
		LGAP	0.277	0.957	14.603	0.206	0.957
	Stacking *(ADA + LGB)*	ADLG	0.339	0.937	25.348	0.253	0.935
	Hybrid ensemble *ADA + LGB+OOA*	ALOA	0.250	0.967	15.934	0.186	0.965
	Hybrid ensemble *ADA + LGB+APO*	ALAP	0.192	0.980	16.097	0.143	0.979
Test	Single models	ADA	0.334	0.944	29.318	0.267	0.936
		LGB	0.382	0.922	35.180	0.306	0.916
	Hybrid models	ADOA	0.305	0.966	23.194	0.244	0.947
		ADAP	0.233	0.971	25.172	0.186	0.969
		LGOA	0.345	0.951	26.711	0.277	0.932
		LGAP	0.301	0.955	22.100	0.241	0.948
	Stacking *(ADA + LGB)*	ADLG	0.312	0.955	25.948	0.250	0.945
	Hybrid ensemble *ADA + LGB+OOA*	ALOA	0.282	0.977	23.878	0.226	0.955
	Hybrid ensemble *ADA + LGB+APO*	ALAP	0.191	0.982	15.611	0.153	0.979

Table 7 compares measured solubility values with those predicted by the different models across training, validation, and test phases using descriptive statistics (minimum, mean, maximum, standard deviation, and skewness). This analysis offers a deeper insight into how well the models capture the underlying data distribution beyond conventional error metrics. In the training phase, most models reproduced the mean solubility values (≈ 1.79–1.85) close to the measured reference (1.818). ADA and LGB slightly underestimated the maximum values (8.122 and 7.491, respectively), whereas ALAP (8.494) and ADAP (8.677) provided closer approximations to the experimental upper range (9.050). Standard deviations across models were marginally lower than measured values, suggesting that while variability was well captured, extreme fluctuations were slightly smoothed by the predictive models. During validation, similar trends were observed. The predicted means remained tightly aligned with the measured value (1.343), with deviations less than 0.08 across all models.

**Table 7:** Statistical metrics employed for model comparison.

Phase	Models		Properties				
			Min	Mean	Max	St. Dev	Skewness
Train	Measured		0.019	1.818	9.050	1.951	1.591
	Predicted	*ADA*	0.031	1.790	8.122	1.866	1.642
		*ADOA*	0.030	1.851	8.135	1.782	1.540
		*ADAP*	0.029	1.827	8.677	1.907	1.639
		*LGB*	0.010	1.787	7.491	1.834	1.409
		*LGOA*	0.013	1.781	8.117	1.846	1.528
		*LGAP*	0.014	1.790	8.309	1.879	1.534
		*ADLG*	0.028	1.789	7.808	1.836	1.546
		*ALOA*	0.030	1.816	8.126	1.807	1.538
		*ALAP*	0.029	1.809	8.494	1.886	1.596
Validation	Measured		0.042	1.343	4.290	1.331	0.827
	Predicted	*ADA*	0.069	1.407	4.908	1.417	1.041
		*ADOA*	0.065	1.420	4.517	1.307	0.894
		*ADAP*	0.066	1.388	4.428	1.328	0.902
		*LGB*	0.012	1.348	4.389	1.266	0.877
		*LGOA*	0.066	1.404	4.612	1.326	0.883
		*LGAP*	0.065	1.373	4.486	1.307	0.807
		*ADLG*	0.041	1.378	4.650	1.330	0.969
		*ALOA*	0.065	1.412	4.564	1.305	0.870
		*ALAP*	0.065	1.381	4.457	1.308	0.845
Test	Measured		0.030	1.249	5.153	1.323	1.475
	Predicted	*ADA*	0.049	1.232	4.812	1.171	1.520
		*ADOA*	0.047	1.285	4.183	1.123	1.185
		*ADAP*	0.046	1.299	5.504	1.344	1.640
		*LGB*	0.038	1.240	5.313	1.177	1.804
		*LGOA*	0.047	1.171	5.021	1.124	1.776
		*LGAP*	0.046	1.259	5.222	1.182	1.659
		*ADLG*	0.050	1.236	5.061	1.160	1.652
		*ALOA*	0.047	1.228	4.599	1.113	1.423
		*ALAP*	0.046	1.279	5.364	1.252	1.660

Notably, ALAP and LGAP offered the closest reproductions of both the mean (1.381 and 1.373, respectively) and skewness (0.845 and 0.807, respectively, compared to 0.827). This indicates that APO-driven and OOA-driven hybrids maintained not only accuracy but also statistical fidelity to the distribution of experimental solubility values. In the test phase, distributional alignment remained strong. ALAP (mean = 1.279, skewness = 1.660) reproduced the overall data structure with high fidelity, closely approximating the measured distribution (mean = 1.249, skewness = 1.475). Compared to single learners, hybrid ensembles better captured both the central tendency and tail behaviour, although slight overestimations in maximum values persisted (e.g., ADAP = 5.504 vs. measured = 5.153). Importantly, the lower standard deviations observed in most models relative to the measured data suggest reduced prediction noise and improved stability.

**Fig. 4:**
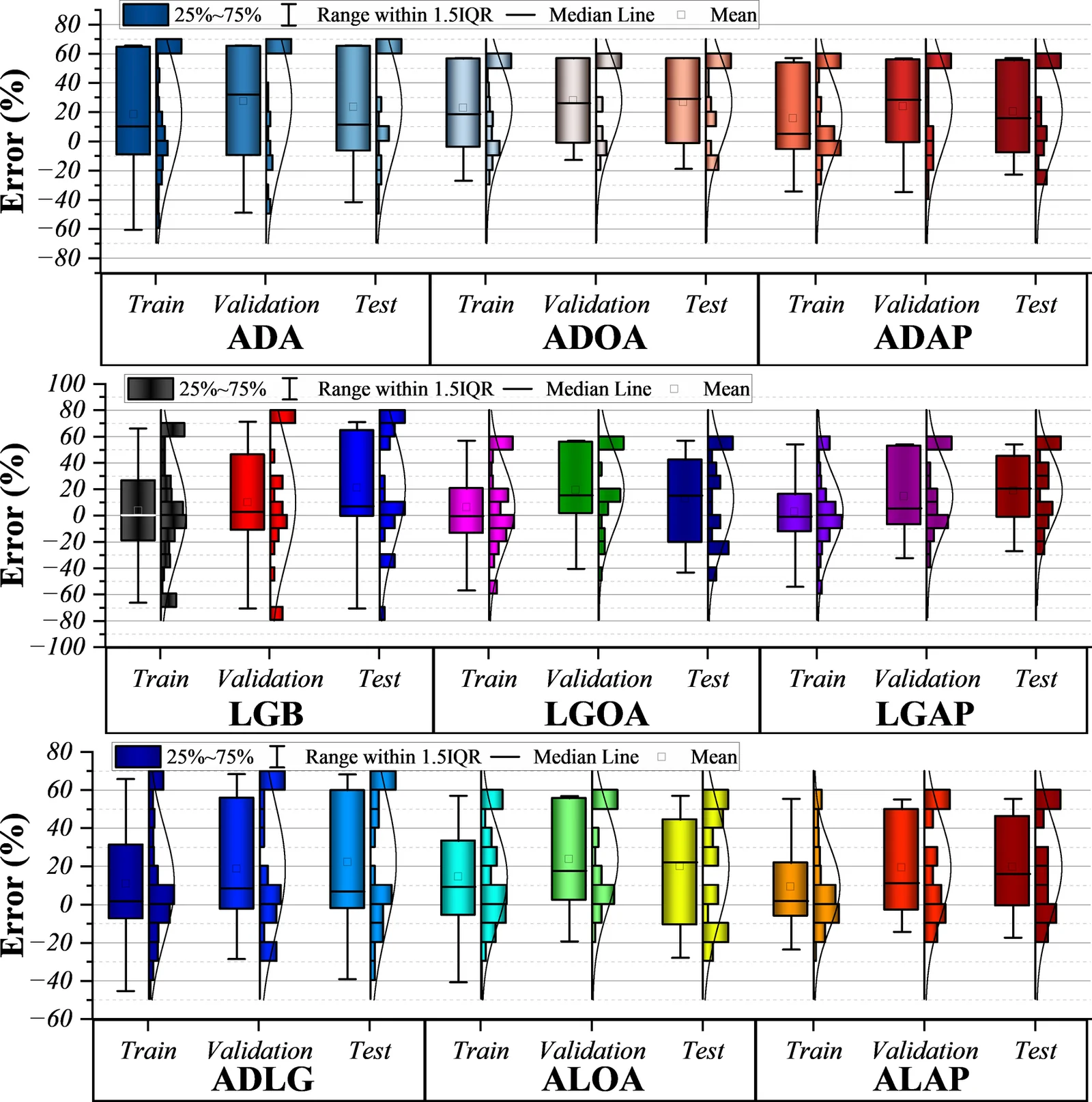
Box normal plot for error analysis, providing a general visualization of error distribution.

Figure 4 shows the distribution of prediction errors across all models. Single learners (ADA, LGB) displayed wide error spreads and frequent outliers, confirming their limited reliability. Hybrid models (ADAP, LGAP) reduced variability and centred the error distribution closer to zero, demonstrating improved generalization. Stacking (ADLG) achieved moderate gains but remained less stable than optimized hybrids. By contrast, hybrid ensembles, especially ALAP, consistently produced compact and symmetric error distributions with minimal bias in both validation and test phases. This indicates not only higher accuracy but also robust and reproducible predictions. From a practical standpoint, such stability is crucial for pharmaceutical applications: models like ALAP minimize the risk of large deviations in solubility prediction, enabling more reliable formulation design and process optimization.

To obtain a fair and integrated ranking of the predictive models, the Technique for Order Preference by Similarity to Ideal Solution (TOPSIS) was employed as a multi-criteria decision-making method. In this study, Root Mean Square Error (RMSE) and coefficient of determination (R²) were selected as decision criteria because they provide complementary information: RMSE evaluates the magnitude of prediction error, whereas R² measures the proportion of variance in the experimental data explained by the model. These two indicators are widely used and represent both accuracy and goodness-of-fit, thereby ensuring balanced model assessment. In the TOPSIS framework, RMSE was treated as a cost criterion to be minimized, and R² was considered a benefit criterion to be maximized. The normalized decision matrix was constructed from these criteria, and the distances to the ideal and negative-ideal solutions were computed to determine the relative closeness of each model to the ideal solution. The final TOPSIS score was then used to rank the competing models.

**Fig. 5:**
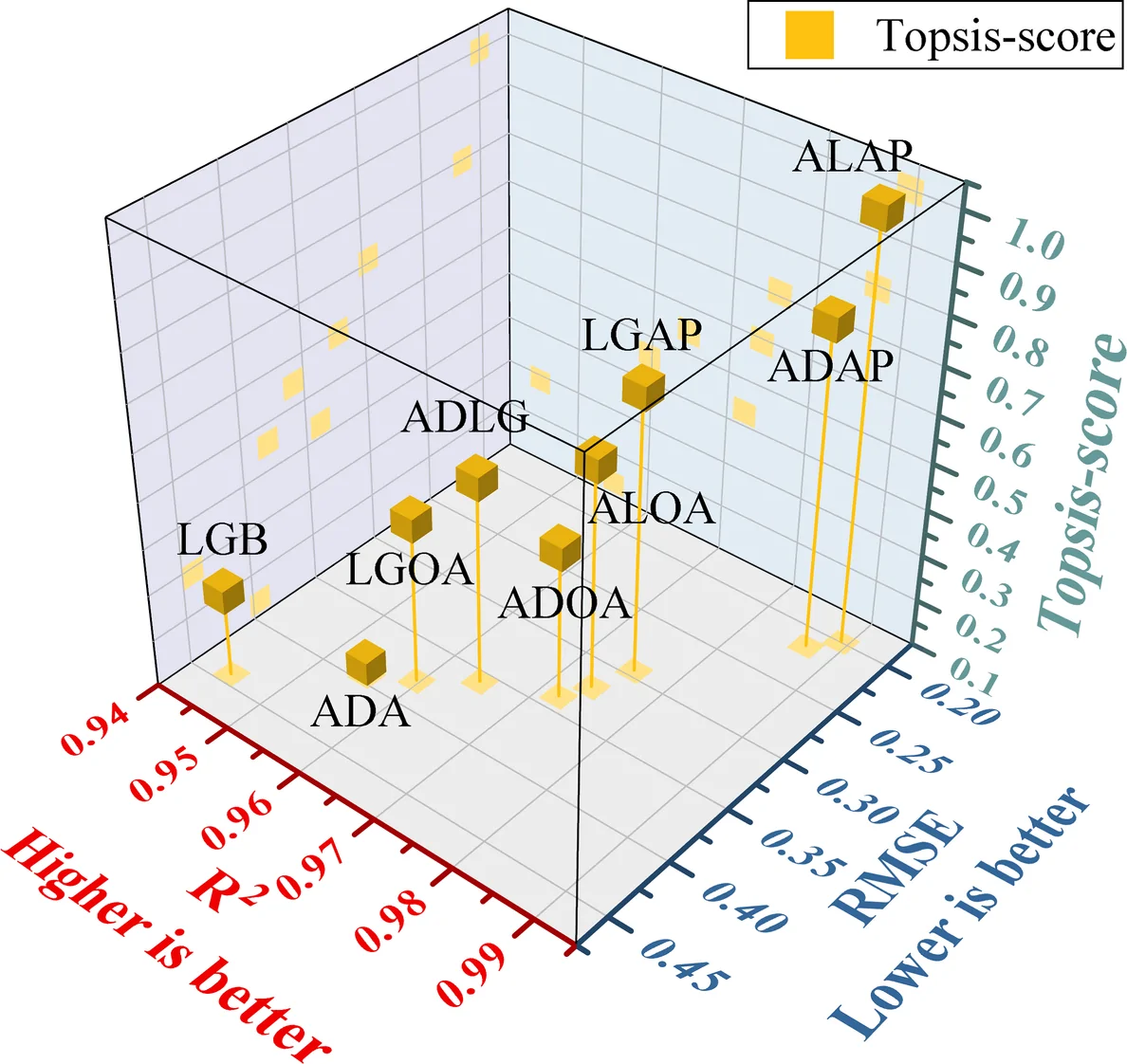
Topsis analyses for the developed models.

As illustrated in Fig. 5, conventional base learners (ADA and LGB) consistently occupied the lower spectrum of the TOPSIS scale, highlighting their limited capacity to balance accuracy and error minimization. Intermediate improvements were observed in optimization-driven hybrids (ADOA, LGOA, ALOA) and stacked models (ADLG), although their TOPSIS scores remained below 0.6, reflecting moderate but inconsistent predictive stability. By contrast, the advanced hybrids ADAP, LGAP, and particularly ALAP, achieved substantially higher scores (> 0.8), with ALAP reaching a near-ideal value of 1.0. This confirms its superior ability to simultaneously maximize predictive accuracy and minimize error, thereby offering a more reliable trade-off across evaluation criteria. Such multi-criteria validation is particularly valuable in real-world pharmaceutical applications, where models must not only demonstrate statistical accuracy but also operational robustness under variable experimental conditions. The consistent superiority of ALAP underscores its suitability as a decision-support tool for accelerating drug solubility prediction, optimizing formulation design, and reducing experimental costs in early-stage drug development.

**Fig. 6:**
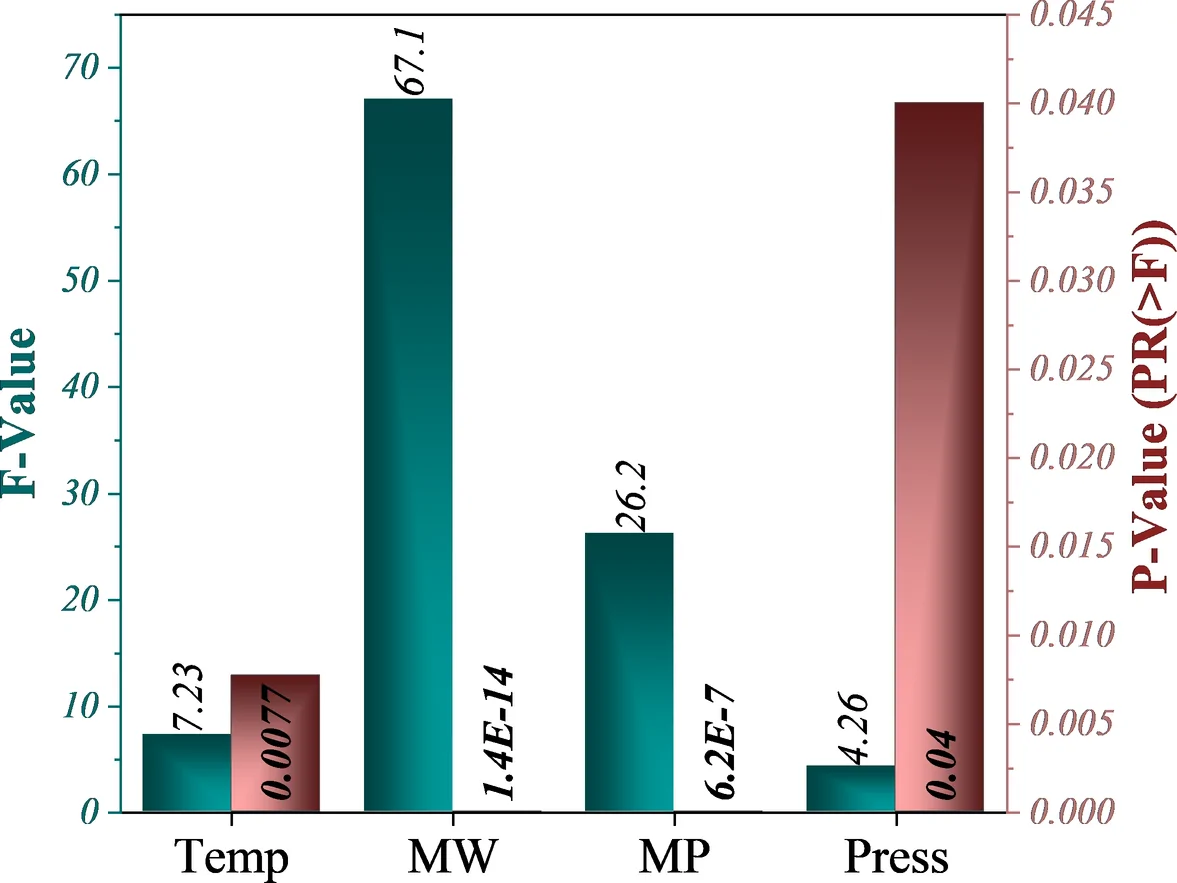
2Ys column plot for ANOVA sensitivity analysis, showing the relative influence of input factors on model performance.

The ANOVA sensitivity analysis highlights the relative contribution of molecular and thermodynamic inputs to solubility prediction (Fig. 6). Molecular weight (MW) exhibited the highest statistical influence, followed by melting point (MP), temperature, and pressure. This ranking reflects the multi-compound nature of the dataset, which spans a wide molecular weight range (157.1–926.09 g·mol⁻¹) and diverse solid-state properties across structurally different pharmaceuticals. In contrast, temperature and pressure vary within comparatively narrower ranges for each compound. Consequently, inter-compound physicochemical variability contributes more strongly to the observed variance in solubility than intra-compound thermodynamic variation in the data-driven sensitivity analysis. This result does not contradict thermodynamic theory, wherein temperature and pressure are the primary state variables governing solubility for a given compound. Instead, MW and MP act as surrogate descriptors for lattice energy, molecular cohesion, and intermolecular interactions, which strongly influence dissolution in supercritical CO₂ across chemically distinct compounds. Thus, their dominance reflects statistical variance within the dataset rather than a replacement of thermodynamic driving forces. Although the present input space includes thermodynamically meaningful variables (temperature and pressure) and key molecular properties (MW and MP), explicit structural descriptors such as aromaticity, ring count, and functional group composition were not included due to inconsistent reporting across experimental sources. Incorporation of such descriptors could further improve mechanistic consistency and interpretability of the models. Future work will extend the framework by integrating cheminformatics-based structural features to achieve closer alignment with molecular thermodynamic behavior.

**Fig. 7:**
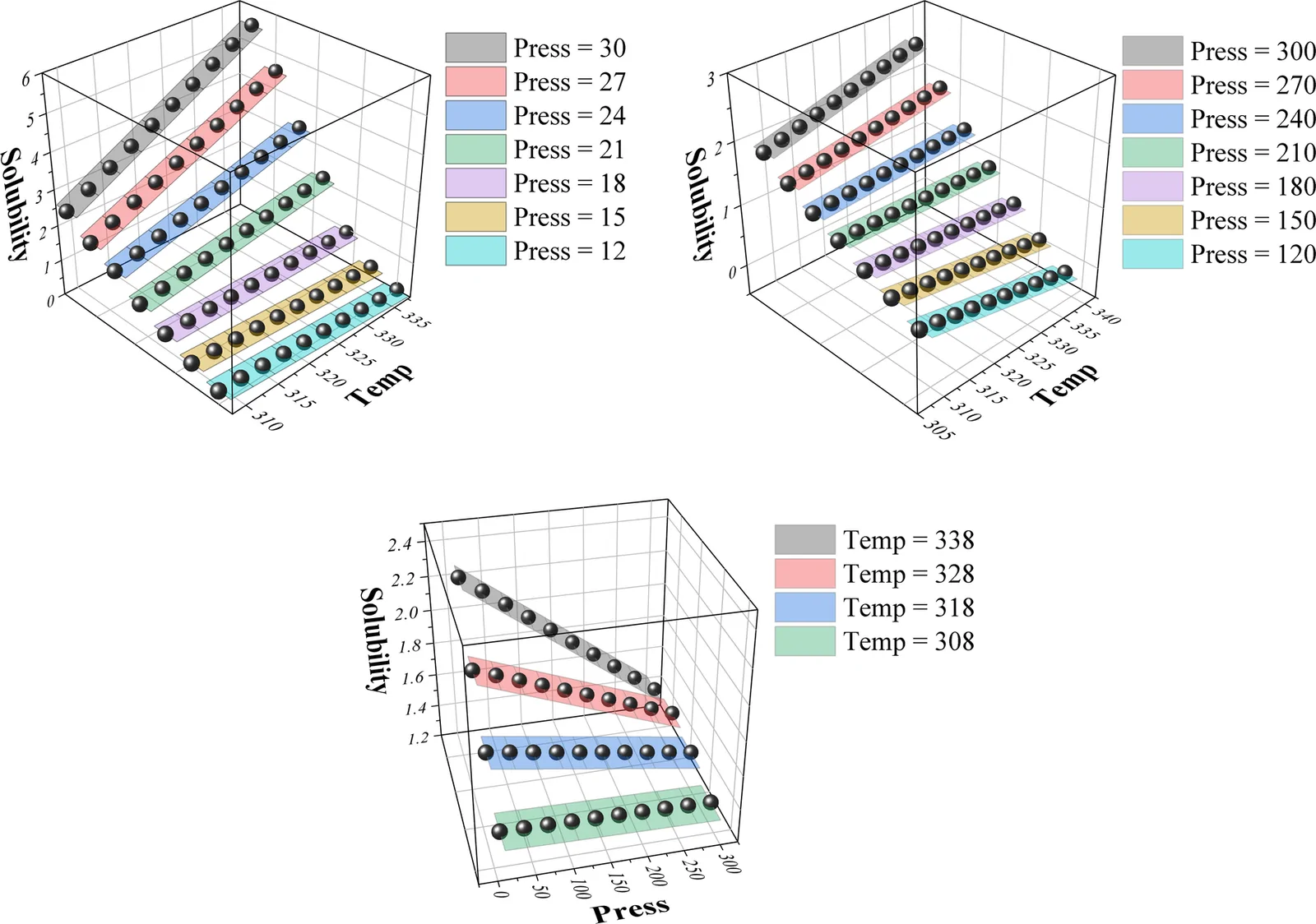
Three-dimensional tendency plots for the combined effects of temperature and pressure on solubility.

The tendency plots (Fig. 7) further reinforce the outcomes of the ANOVA sensitivity analysis by visually capturing the parametric influence of temperature and pressure on solubility. Across all scenarios, solubility exhibited a direct, positive correlation with temperature, with higher temperatures consistently enhancing solubility, regardless of the pressure conditions. This aligns with the earlier observation that temperature, although less dominant than molecular weight and melting point, still plays a significant role in modulating system behaviour. Pressure, in contrast, displayed a more gradual but steady impact, particularly at elevated temperatures where the slope of the solubility increase became more pronounced.

The bottom panel highlights that, at constant temperatures, solubility improves with increasing pressure, although the magnitude of the change is less substantial compared to the effect of temperature. These results highlight the synergistic interaction between temperature and pressure, where temperature serves as the primary driving force, while pressure fine-tunes the solubility outcome. From a practical standpoint, this tendency suggests that optimizing solubility in supercritical systems requires prioritizing temperature adjustments while employing pressure as a complementary control parameter. Such insights are valuable for process engineers aiming to design energy-efficient and high-yield operations, as they highlight the conditions under which operational flexibility can be achieved without compromising performance.

To further validate the robustness of the predictive models, the Wilcoxon signed-rank test was employed to compare predicted and measured values and assess whether systematic differences exist (Table 8). Unlike parametric tests, the Wilcoxon test does not assume normality of the data and is therefore suitable for paired prediction–observation comparisons. The test statistic (* W*) is computed from the signed ranks of the absolute differences between paired values and is used to derive the corresponding p-value. A p-value greater than 0.05 indicates that no statistically significant bias exists between predicted and experimental values at the 95% confidence level. The results reveal that most models (ADA, LGB, LGAP, LGOA, ADLG, and ALAP) exhibited p-values greater than 0.05, indicating no statistically significant deviation between predicted and experimental data. This outcome underscores their reliability and generalization capability across varying data partitions. Notably, the ADOA and ALOA models yielded p-values of 0.0003 and 0.0096, respectively, both of which are below the 0.05 threshold, suggesting significant differences between the predicted and observed results. While these models performed competitively in terms of error-based metrics, the Wilcoxon test highlights limitations in their consistency under certain validation scenarios. In contrast, ADAP and ALAP demonstrated a balance between predictive accuracy and statistical robustness, achieving strong performance metrics with no significant bias relative to experimental measurements.

**Table 8:** Results of the Wilcoxon signed-rank test, including the test statistic (*W*) and corresponding p-values for each predictive model.

Models	Parameter	
	Statistical	P-value
ADAP	13106	0.0773
ADOA	11747	0.0003
ADA	15057	0.4464
LGAP	15867.5	0.9508
LGOA	15640	0.7963
LGB	15333	0.6008
ALAP	14369	0.1753
ALOA	12941	0.0096
ADLG	14413	0.1877

To further validate the reliability of the proposed framework, its predictive performance was compared with representative thermodynamic and artificial intelligence-based models reported in the literature. Table 9 summarizes typical performance indicators achieved by equation-of-state (EoS) correlations, ANFIS-based models, and recent machine learning approaches for drug solubility prediction in supercritical CO₂. Classical thermodynamic models, such as Peng–Robinson and Soave–Redlich–Kwong equations of state, generally provide reasonable trend prediction but require compound-specific parameter fitting and often yield lower accuracy when applied across chemically diverse drugs (typical R² < 0.95 and AARD > 10%). ANFIS-based models reported for individual compounds exhibit improved accuracy (R² ≈ 0.97–0.99), although their performance is usually limited to narrow compound classes and restricted operating ranges. More recent AI-based approaches, including ensemble regressors and hybrid deep learning models, have achieved higher predictive accuracy (R² ≈ 0.98–0.99) but often rely on extensive descriptor sets or compound-specific training. In comparison, the proposed APO-driven hybrid ensemble (ALAP) achieved an R² of 0.982 and an RMSE of 0.191 on an independent test set covering seven chemically distinct pharmaceuticals. Unlike compound-specific thermodynamic correlations or single-compound ANFIS models, the present framework was trained on a multi-compound dataset and demonstrated consistent performance across different molecular structures and thermodynamic conditions. This confirms that the developed model not only matches but in several cases exceeds the predictive capability of previously reported thermodynamic and AI-based approaches, while offering broader applicability and improved generalization.

**Table 9:** Comparison of the proposed model with selected thermodynamic and AI-based solubility prediction models from literature.

Study	Method	Dataset scope	Performance
Sodeifian et al.^18^	PR, SRK (EoS)	Single drug (Gemcitabine)	R^2^ ≈ 0.96
Laggoune et al.^19^	EoS (SRK)	Single drug (Sirolimus)	R^2^ ≈ 0.98
Zhu et al.^22^	ANFIS	Single drug (Busulfan)	R^2^ ≈ 0.99
Bahrami et al.^8^	ANFIS	Multiple drugs	RMSE ≈ 0.26
Damansabz et al.^23^	GWO-DE-LSTM	Multiple drugs	R^2^ ≈ 0.93
Present study	ALAP (ADA + LGB+APO)	Seven drugs (252 samples)	R^2^ = 0.982, RMSE = 0.191

## Conclusion

This work innovatively designed and extensively evaluated superior machine learning architectures to accurately model pharmaceutical compound solubility in supercritical CO₂ with the overall intention of assisting greener and more efficient pharmaceutical procedure construction. Two baseline booster models, Adaptive Boosting (ADA) and Light Gradient Boosting (LGB), were used as the baseline, and their predictive might was improved by combining them with metaheuristic optimization algorithms like the Osprey Optimization Algorithm (OOA) and the Artificial Protozoa Optimizer (APO). Additional ensemble methods, such as stacking (ADLG) and hybrid ensembles (ALOA and ALAP), were constructed as an effort towards combining the strength of individual learners. Experimentations conirmed that hybrid ensembles outperformed single models as well as classical stacking at each train, validation, as well as test phases. In particular, the ALAP paradigm (ADA + LGB+APO) attained the most stable outcome delivering an RMSE of 0.191, R² of 0.982, as well as MDAPE of 15.6 on the test set. Integration of APO provided particularly advantageous effects towards optimisation of hyperparameters as well as enhancing generalisability. Statistical analyses, such as ANOVA-based sensitivity analysis, revealed that the largest contributing factor towards solubility prediction was that of molecular weight followed by that of melting point, temperature, as well as pressure. These findings were also confirmed by way of descriptive statistics as well as the Wilcoxon signed-rank test verifying the strength as well as consistency of the proposed models. Apart from accuracy, the tendency plots provided mechanistic evidence wherein temperature as well as pressure execute interactions coupled with compound-speci c properties towards altering solubility. Such interpretability not only merely justifies the predictive framework but also fosters its pragmatic worth towards pharmaceutical design by delivering more sustainable as well as resource-e cient formulation strategies. In short, this paper offers an effective, generalisable method integrating boosting-based learners, bio-inspired optimizers, and multi-criterion verification towards overcoming the hitherto bugbear of solubility prediction. Through reducing reliance on tedious experimental tests, the proposed framework holds the promise of speeding up processes of pharmaceutical development pipelines, decreasing use of solvents, as well as propelling towards the ideal aim of environmentally sustainable pharma processes. Potential follow-up study could elongate such a strategy by integrating larger molecular datasets coupled with investigating deeper learning architectures as well as extending its application towards other physicochemical properties crucial for drugs exploration as well as formulation.

## Supplementary Information

Below is the link to the electronic supplementary material.

Supplementary Material 1

Supplementary Material 2

## Data Availability

The datasets used and analysed in this study are provided as Supplementary Material (Supplementary Data S1).

## Code availability

The custom Python codes used for model development, hyperparameter optimization, ensemble learning, and statistical analysis in this study are provided as Supplementary Material (Supplementary Code S2).
