# Supplementary material for: Automated machine-learning framework for predicting drug solubility in supercritical CO2 for sustainable process development
Source: Sci Rep. 2026 Jun 25;16:28594. doi: 10.1038/s41598-026-59449-z (PMC13575184; doi:10.1038/s41598-026-59449-z)
Supplement: Supplementary file 1 [file 41598_2026_59449_MOESM1_ESM.pdf]

S2: Developed python codes for article

---

1. ADA.py — AdaBoost Regressor

python

```
import numpy as np
import sklearn.ensemble as en
from sklearn.tree import DecisionTreeRegressor as dtr

X=np.loadtxt('input.txt')
Y=np.loadtxt('target.txt')
size_Y = len(Y)
size_tr = round(size_Y*0.8)
size_te = size_Y-size_tr

X_tr = X[:size_tr]
X_te = X[size_tr:]
Y_tr = Y[:size_tr]
Y_te = Y[size_tr:]

Y_tr = np.array(Y_tr).reshape(size_tr)
Y_te = np.array(Y_te).reshape(size_te)

weak_learner = dtr(max_leaf_nodes=7)
net = en.AdaBoostRegressor(base_estimator=weak_learner, n_estimators=7)

net.fit(X_tr, Y_tr)
preds_tr = net.predict(X_tr)
```

```
preds_te = net.predict(X_te)
y = np.hstack([Y_tr, Y_te])
preds = np.hstack([preds_tr, preds_te])

ytr = preds
N = len(Y)
S1=S2=S4=S5=S6=S7=R1=R2=R3=M=Z=0

for i in range(N):
    S1 = (ytr[i]-Y[i])**2
    M = M+S1
    S2 = abs(ytr[i]-Y[i])
    Z = Z+S2
    S4 = (Y[i]-np.mean(Y))*(ytr[i]-np.mean(ytr))
    R1 = S4+R1
    S5 = (ytr[i]-np.mean(ytr))**2
    R2 = R2+S5
    S6 = (Y[i]-np.mean(Y))**2
    R3 = R3+S6
    S7 = abs((ytr[i]-Y[i])/ytr[i])

MSE = M/N
RMSE = np.sqrt(MSE)
print(RMSE)
R_2 = (R1/np.sqrt(R2*R3))**2
print("R2:", R_2)
MAPE = S7/N
```

---

2. LGBM.py — LightGBM Regressor

```python
import numpy as np
import lightgbm as lgbm

X=np.loadtxt('input.txt')
Y=np.loadtxt('target.txt')
size_Y = len(Y)
size_tr = round(size_Y*0.7)
size_te = size_Y-size_tr

X_tr = X[:size_tr]
X_te = X[size_tr:]
Y_tr = Y[:size_tr]
Y_te = Y[size_tr:]

Y_tr = np.array(Y_tr).reshape(size_tr)
Y_te = np.array(Y_te).reshape(size_te)

net = lgbm.LGBMRegressor()
net.fit(X_tr, Y_tr)

preds_tr = net.predict(X_tr)
preds_te = net.predict(X_te)

Y = np.hstack([Y_tr, Y_te])
```

```
preds = np.hstack([preds_tr, preds_te])
ytr = preds

N = len(Y)
S1=S2=S4=S5=S6=S7=R1=R2=R3=M=Z=0

for i in range(N):
    S1 = (ytr[i]-Y[i])**2
    M = M+S1
    S2 = abs(ytr[i]-Y[i])
    Z = Z+S2
    S4 = (Y[i]-np.mean(Y))*(ytr[i]-np.mean(ytr))
    R1 = S4+R1
    S5 = (ytr[i]-np.mean(ytr))**2
    R2 = R2+S5
    S6 = (Y[i]-np.mean(Y))**2
    R3 = R3+S6
    S7 = abs((ytr[i]-Y[i])/ytr[i])

MSE = M/N
RMSE = np.sqrt(MSE)
print(RMSE)
R_2 = (R1/np.sqrt(R2*R3))**2
print(R_2)
MAPE = S7/N
```

---

3. K_Fold_Reg.py — K-Fold Cross-Validation

```python
import numpy as np
import xgboost as xgb
import sklearn.metrics as met
from sklearn.model_selection import KFold

X=np.loadtxt('input.txt')
Y=np.loadtxt('target.txt')

kf = KFold(n_splits=10, shuffle=False)
kf.get_n_splits(X)
best_cost = -np.inf
R_2_store = []
RMSE_store = []
MAE_store = []
x_store = []
y_store = []
preds_store = []

net = xgb.XGBRegressor(objective='reg:squarederror')

for i, (train_index, test_index) in enumerate(kf.split(X)):
    X_tr = X[train_index]
    X_te = X[test_index]
    Y_tr = Y[train_index]
    Y_te = Y[test_index]
```

```
net.fit(X_tr, Y_tr)
preds_tr = net.predict(X_tr)
preds_te = net.predict(X_te)
x = np.vstack([X_tr, X_te])
x_store.append(x)
y = np.hstack([Y_tr, Y_te])
y_store.append(y)
preds = np.hstack([preds_tr, preds_te])
preds_store.append(preds)
MSE = met.mean_squared_error(y, preds)
RMSE = np.sqrt(MSE)
RMSE_store.append(RMSE)

S4=S5=S6=R1=R2=R3=Z=0
for i in range(len(y)):
    S4 = (y[i]-np.mean(y))*(preds[i]-np.mean(preds))
    R1 = S4+R1
    S5 = (preds[i]-np.mean(preds))**2
    R2 = R2+S5
    S6 = (y[i]-np.mean(y))**2
    R3 = R3+S6
    Z += abs(preds[i] - y[i])
R_2 = (R1/np.sqrt(R2*R3))**2
R_2_store.append(R_2)
MAE = Z / len(y)
MAE_store.append(MAE)
if R_2 > best_cost:
```

```
        best_cost = R_2
        best_R_2 = R_2
        X_tr_best = X_tr
        X_te_best = X_te
        Y_tr_best = Y_tr
        Y_te_best = Y_te
```

---

4. OOA_ADAR.py — Osprey Optimization + AdaBoost

python

```
import numpy as np
import sklearn.ensemble as en
from sklearn.tree import DecisionTreeRegressor as dtr
from ypstruct import structure
import sklearn.metrics as met


def cost_function(pop, X_tr, X_te, Y_tr, Y_te):
    weak_learner = dtr(max_leaf_nodes=200)
    net = en.AdaBoostRegressor(base_estimator=weak_learner,
                               n_estimators=int(pop[0]), learning_rate=pop[1])
    net.fit(X_tr, Y_tr)
    preds_tr = net.predict(X_tr)
    preds_te = net.predict(X_te)
    y = np.hstack([Y_tr, Y_te])
    preds = np.hstack([preds_tr, preds_te])
    MSE = met.mean_squared_error(y, preds)
    RMSE = np.sqrt(MSE)
    POP = structure(cost=RMSE, pre=preds, net=net)
```

```
    return POP.cost, POP.pre

def levy_flight(dim, beta=1.5):
    sigma = (np.math.gamma(1+beta) * np.sin(np.pi*beta/2) /
             (np.math.gamma((1+beta)/2) * beta * 2**((beta-1)/2)))**(1/beta)
    u = np.random.randn(dim) * sigma
    v = np.random.randn(dim)
    return u / (np.abs(v)**(1/beta))

def Osprey_optimizer(SearchAgents, Max_iterations, lowerbound, upperbound,
                     dimension, X_tr, X_te, Y_tr, Y_te):
    X = np.random.uniform(lowerbound, upperbound, (SearchAgents, dimension))
    fitness = np.zeros(SearchAgents)
    POP = [structure(cost=None, pre=None) for _ in range(SearchAgents)]

    for i in range(SearchAgents):
        fitness[i], POP[i].pre = cost_function(X[i], X_tr, X_te, Y_tr, Y_te)
        POP[i].cost = fitness[i]

    best_idx = np.argmin(fitness)
    Best_pos = X[best_idx].copy()
    Best_score = fitness[best_idx]
    Best_pre = POP[best_idx].pre
    HO_curve = np.zeros(Max_iterations)

    for t in range(Max_iterations):
        r = 0.2 * (1 - t / Max_iterations)
```

```
    for i in range(SearchAgents):
      Phase1 = np.random.choice([0, 1], p=[0.5, 0.5])
      if Phase1 == 0:
        candidates = [c for c in range(SearchAgents) if c != i]
        k = np.random.choice(candidates)
        F = 1.5 * np.random.rand() + 0.5
        new_position = np.clip(X[i] + F * (X[k] - X[i]), lowerbound, upperbound)
        new_fitness, new_pre = cost_function(new_position, X_tr, X_te, Y_tr, Y_te)
        if new_fitness < fitness[i]:
          X[i], fitness[i], POP[i].pre, POP[i].cost = new_position, new_fitness, new_pre,
new_fitness
          if new_fitness < Best_score:
            Best_score, Best_pos, Best_pre = new_fitness, new_position.copy(), new_pre
      else:
        SF = 0.2 + 0.8 * (t / Max_iterations)
        for j in range(dimension):
          if np.random.rand() < SF:
            new_position = X[i].copy()
            new_position[j] = np.clip(
              X[i][j] + r * (upperbound - lowerbound) * np.random.randn(),
              lowerbound, upperbound)
            new_fitness, new_pre = cost_function(new_position, X_tr, X_te, Y_tr, Y_te)
            if new_fitness < fitness[i]:
              X[i], fitness[i], POP[i].pre, POP[i].cost = new_position, new_fitness, new_pre,
new_fitness
              if new_fitness < Best_score:
```

```
                    Best_score, Best_pos, Best_pre = new_fitness, new_position.copy(),
new_pre

        for i in range(SearchAgents):
            if np.random.rand() < 0.1:
                new_position = np.clip(Best_pos + levy_flight(dimension) * (Best_pos - X[i]),
                                       lowerbound, upperbound)
                new_fitness, new_pre = cost_function(new_position, X_tr, X_te, Y_tr, Y_te)
                if new_fitness < fitness[i]:
                    X[i], fitness[i], POP[i].pre, POP[i].cost = new_position, new_fitness, new_pre,
new_fitness
                    if new_fitness < Best_score:
                        Best_score, Best_pos, Best_pre = new_fitness, new_position.copy(), new_pre

        HO_curve[t] = Best_score
        print(f'Iteration {t+1}/{Max_iterations}, Best RMSE = {Best_score:.6f}')

    return Best_score, Best_pos, Best_pre, HO_curve

XX = np.loadtxt('input.txt')
Y = np.loadtxt('target.txt')
size_Y = len(Y)
size_tr = round(size_Y*0.8)
size_te = size_Y - size_tr
X_tr, X_te = XX[:size_tr], XX[size_tr:]
Y_tr = Y[:size_tr].reshape(size_tr)
Y_te = Y[size_tr:].reshape(size_te)
```

```
dimension, lowerbound, upperbound = 2, 1, 999
SearchAgents, Max_iterations = 50, 200

Best_score, Best_pos, Best_pre, HO_curve = Osprey_optimizer(
    SearchAgents, Max_iterations, lowerbound, upperbound, dimension,
    X_tr, X_te, Y_tr, Y_te)

ytr = Best_pre
N = len(Y)
MSE = np.mean((ytr - Y)**2)
RMSE = np.sqrt(MSE)
MAE = np.mean(np.abs(ytr - Y))
R2 = np.corrcoef(Y, ytr)[0, 1]**2
with np.errstate(divide='ignore', invalid='ignore'):
    MAPE = np.mean(np.abs((Y - ytr) / ytr)) * 100

print(f"RMSE: {RMSE:.6f}, MSE: {MSE:.6f}, MAE: {MAE:.6f}, R²: {R2:.6f}, MAPE: {MAPE:.2f}%")
```

---

5. OOA_LGBMR.py — Osprey Optimization + LightGBM

python

```
import numpy as np
import lightgbm as lgbm
from ypstruct import structure
import sklearn.metrics as met

def cost_function(pop, X_tr, X_te, Y_tr, Y_te):
```

```
    net = lgbm.LGBMRegressor(num_leaves=int(pop[0]+1), max_depth=int(pop[1]),
                             learning_rate=pop[2]/1000, n_estimators=int(pop[3]),
                             boosting_type='dart', max_bin=int(pop[4]*1000))
    net.fit(X_tr, Y_tr)
    preds_tr = net.predict(X_tr)
    preds_te = net.predict(X_te)
    y = np.hstack([Y_tr, Y_te])
    preds = np.hstack([preds_tr, preds_te])
    MSE = met.mean_squared_error(y, preds)
    RMSE = np.sqrt(MSE)
    POP = structure(cost=RMSE, pre=preds, net=net)
    return POP.cost, POP.pre

def levy_flight(dim, beta=1.5):
    sigma = (np.math.gamma(1+beta) * np.sin(np.pi*beta/2) /
          (np.math.gamma((1+beta)/2) * beta * 2**((beta-1)/2)))**(1/beta)
    u = np.random.randn(dim) * sigma
    v = np.random.randn(dim)
    return u / (np.abs(v)**(1/beta))

def Osprey_optimizer(SearchAgents, Max_iterations, lowerbound, upperbound,
                     dimension, X_tr, X_te, Y_tr, Y_te):
    X = np.random.uniform(lowerbound, upperbound, (SearchAgents, dimension))
    fitness = np.zeros(SearchAgents)
    POP = [structure(cost=None, pre=None) for _ in range(SearchAgents)]

    for i in range(SearchAgents):
```

```
        fitness[i], POP[i].pre = cost_function(X[i], X_tr, X_te, Y_tr, Y_te)
        POP[i].cost = fitness[i]

    best_idx = np.argmin(fitness)
    Best_pos, Best_score, Best_pre = X[best_idx].copy(), fitness[best_idx], POP[best_idx].pre
    HO_curve = np.zeros(Max_iterations)

    for t in range(Max_iterations):
        r = 0.2 * (1 - t / Max_iterations)
        for i in range(SearchAgents):
            Phase1 = np.random.choice([0, 1], p=[0.5, 0.5])
            if Phase1 == 0:
                candidates = [c for c in range(SearchAgents) if c != i]
                k = np.random.choice(candidates)
                F = 1.5 * np.random.rand() + 0.5
                new_position = np.clip(X[i] + F * (X[k] - X[i]), lowerbound, upperbound)
                new_fitness, new_pre = cost_function(new_position, X_tr, X_te, Y_tr, Y_te)
                if new_fitness < fitness[i]:
                    X[i], fitness[i], POP[i].pre, POP[i].cost = new_position, new_fitness, new_pre,
new_fitness
                    if new_fitness < Best_score:
                        Best_score, Best_pos, Best_pre = new_fitness, new_position.copy(), new_pre
            else:
                SF = 0.2 + 0.8 * (t / Max_iterations)
                for j in range(dimension):
                    if np.random.rand() < SF:
                        new_position = X[i].copy()
```

```
                        new_position[j] = np.clip(
                            X[i][j] + r * (upperbound - lowerbound) * np.random.randn(),
                            lowerbound, upperbound)
                        new_fitness, new_pre = cost_function(new_position, X_tr, X_te, Y_tr, Y_te)
                        if new_fitness < fitness[i]:
                            X[i], fitness[i], POP[i].pre, POP[i].cost = new_position, new_fitness, new_pre,
new_fitness
                            if new_fitness < Best_score:
                                Best_score, Best_pos, Best_pre = new_fitness, new_position.copy(),
new_pre

        for i in range(SearchAgents):
            if np.random.rand() < 0.1:
                new_position = np.clip(Best_pos + levy_flight(dimension) * (Best_pos - X[i]),
                                       lowerbound, upperbound)
                new_fitness, new_pre = cost_function(new_position, X_tr, X_te, Y_tr, Y_te)
                if new_fitness < fitness[i]:
                    X[i], fitness[i], POP[i].pre, POP[i].cost = new_position, new_fitness, new_pre,
new_fitness
                    if new_fitness < Best_score:
                        Best_score, Best_pos, Best_pre = new_fitness, new_position.copy(), new_pre

        HO_curve[t] = Best_score
        print(f'Iteration {t+1}/{Max_iterations}, Best RMSE = {Best_score:.6f}')

    return Best_score, Best_pos, Best_pre, HO_curve

XX = np.loadtxt('input.txt')
```

```
Y = np.loadtxt('target.txt')
size_Y = len(Y)
size_tr = round(size_Y*0.8)
size_te = size_Y - size_tr
X_tr, X_te = XX[:size_tr], XX[size_tr:]
Y_tr = Y[:size_tr].reshape(size_tr)
Y_te = Y[size_tr:].reshape(size_te)

dimension, lowerbound, upperbound = 5, 1, 999
SearchAgents, Max_iterations = 50, 200

Best_score, Best_pos, Best_pre, HO_curve = Osprey_optimizer(
    SearchAgents, Max_iterations, lowerbound, upperbound, dimension,
    X_tr, X_te, Y_tr, Y_te)

ytr = Best_pre
N = len(Y)
MSE = np.mean((ytr - Y)**2)
RMSE = np.sqrt(MSE)
MAE = np.mean(np.abs(ytr - Y))
R2 = np.corrcoef(Y, ytr)[0, 1]**2
with np.errstate(divide='ignore', invalid='ignore'):
    MAPE = np.mean(np.abs((Y - ytr) / ytr)) * 100

print(f"RMSE: {RMSE:.6f}, MSE: {MSE:.6f}, MAE: {MAE:.6f}, R²: {R2:.6f}, MAPE: {MAPE:.2f}%")
```

---

6. APO_ADAR.py — Artificial Protozoa Optimization + AdaBoost

python

```
import numpy as np
import sklearn.ensemble as en
from sklearn.tree import DecisionTreeRegressor as dtr
from ypstruct import structure
import sklearn.metrics as met


def cost_function(pop, X_tr, X_te, Y_tr, Y_te):
    weak_learner = dtr(max_leaf_nodes=200)
    net = en.AdaBoostRegressor(base_estimator=weak_learner,
                               n_estimators=int(pop[0]), learning_rate=pop[1])
    net.fit(X_tr, Y_tr)
    preds_tr = net.predict(X_tr)
    preds_te = net.predict(X_te)
    y = np.hstack([Y_tr, Y_te])
    preds = np.hstack([preds_tr, preds_te])
    MSE = met.mean_squared_error(y, preds)
    RMSE = np.sqrt(MSE)
    POP = structure(cost=RMSE, pre=preds, net=net)
    return POP.cost, POP.pre


def levy_flight(dim, beta=1.5):
    sigma = (np.math.gamma(1+beta) * np.sin(np.pi*beta/2) /
             (np.math.gamma((1+beta)/2) * beta * 2**((beta-1)/2)))**(1/beta)
    u = np.random.randn(dim) * sigma
    v = np.random.randn(dim)
```

```python
    return u / (np.abs(v)**(1/beta))

def APO_optimizer(SearchAgents, Max_iterations, lowerbound, upperbound,
                  dimension, X_tr, X_te, Y_tr, Y_te):
    X = np.random.uniform(lowerbound, upperbound, (SearchAgents, dimension))
    fitness = np.zeros(SearchAgents)
    POP = [structure(cost=None, pre=None) for _ in range(SearchAgents)]

    for i in range(SearchAgents):
        fitness[i], POP[i].pre = cost_function(X[i], X_tr, X_te, Y_tr, Y_te)
        POP[i].cost = fitness[i]

    best_idx = np.argmin(fitness)
    Best_pos, Best_score, Best_pre = X[best_idx].copy(), fitness[best_idx], POP[best_idx].pre

    w_max, w_min, C, S, T = 0.9, 0.4, 1.5, 0.5, 0.3
    HO_curve = np.zeros(Max_iterations)

    for t in range(Max_iterations):
        w = w_max - (w_max - w_min) * (t / Max_iterations)
        for i in range(SearchAgents):
            candidates = [c for c in range(SearchAgents) if c != i]
            r1, r2 = np.random.choice(candidates, 2, replace=False)
            rand_val = np.random.rand()
            if rand_val < T:
                new_position = X[i] + levy_flight(dimension) * (X[r1] - X[r2])
            elif rand_val < T + S:
```

```
            phi = np.random.uniform(-1, 1, dimension)
            new_position = X[i] + w * phi * (Best_pos - X[i]) + \
                        np.random.rand() * (X[r1] - X[r2])
        else:
            A = 2 * np.random.rand() - 1
            new_position = X[i] + C * np.random.rand(dimension) * \
                        (A * (Best_pos - X[i]) + (1-A) * (X[r1] - X[r2]))
        new_position = np.clip(new_position, lowerbound, upperbound)
        new_fitness, new_pre = cost_function(new_position, X_tr, X_te, Y_tr, Y_te)
        if new_fitness < fitness[i]:
            X[i], fitness[i], POP[i].pre, POP[i].cost = new_position, new_fitness, new_pre,
new_fitness
            if new_fitness < Best_score:
                Best_score, Best_pos, Best_pre = new_fitness, new_position.copy(), new_pre

    if t % 20 == 0 and t > 0:
        n_restart = max(1, int(SearchAgents * 0.1))
        for idx in np.argsort(fitness)[-n_restart:]:
            X[idx] = np.random.uniform(lowerbound, upperbound, dimension)
            fitness[idx], POP[idx].pre = cost_function(X[idx], X_tr, X_te, Y_tr, Y_te)
            POP[idx].cost = fitness[idx]

    HO_curve[t] = Best_score
    print(f'Iteration {t+1}/{Max_iterations}, Best RMSE = {Best_score:.6f}')

return Best_score, Best_pos, Best_pre, HO_curve
```

```
XX = np.loadtxt('input.txt')
Y = np.loadtxt('target.txt')
size_Y = len(Y)
size_tr = round(size_Y*0.8)
size_te = size_Y - size_tr
X_tr, X_te = XX[:size_tr], XX[size_tr:]
Y_tr = Y[:size_tr].reshape(size_tr)
Y_te = Y[size_tr:].reshape(size_te)

dimension, lowerbound, upperbound = 2, 1, 999
SearchAgents, Max_iterations = 50, 200

Best_score, Best_pos, Best_pre, HO_curve = APO_optimizer(
    SearchAgents, Max_iterations, lowerbound, upperbound, dimension,
    X_tr, X_te, Y_tr, Y_te)

ytr = Best_pre
N = len(Y)
MSE = np.mean((ytr - Y)**2)
RMSE = np.sqrt(MSE)
MAE = np.mean(np.abs(ytr - Y))
R2 = np.corrcoef(Y, ytr)[0, 1]**2
with np.errstate(divide='ignore', invalid='ignore'):
    MAPE = np.mean(np.abs((Y - ytr) / ytr)) * 100

print(f"RMSE: {RMSE:.6f}, MSE: {MSE:.6f}, MAE: {MAE:.6f}, R²: {R2:.6f}, MAPE: {MAPE:.2f}%")
```

7. APO_LGBMR.py — Artificial Protozoa Optimization + LightGBM

python

```
import numpy as np
import lightgbm as lgbm
from ypstruct import structure
import sklearn.metrics as met


def cost_function(pop, X_tr, X_te, Y_tr, Y_te):
    net = lgbm.LGBMRegressor(num_leaves=int(pop[0]+1), max_depth=int(pop[1]),
                             learning_rate=pop[2]/1000, n_estimators=int(pop[3]),
                             boosting_type='dart', max_bin=int(pop[4]*1000))
    net.fit(X_tr, Y_tr)
    preds_tr = net.predict(X_tr)
    preds_te = net.predict(X_te)
    y = np.hstack([Y_tr, Y_te])
    preds = np.hstack([preds_tr, preds_te])
    MSE = met.mean_squared_error(y, preds)
    RMSE = np.sqrt(MSE)
    POP = structure(cost=RMSE, pre=preds, net=net)
    return POP.cost, POP.pre


def levy_flight(dim, beta=1.5):
    sigma = (np.math.gamma(1+beta) * np.sin(np.pi*beta/2) /
             (np.math.gamma((1+beta)/2) * beta * 2**((beta-1)/2)))**(1/beta)
    u = np.random.randn(dim) * sigma
    v = np.random.randn(dim)
```

```python
    return u / (np.abs(v)**(1/beta))

def APO_optimizer(SearchAgents, Max_iterations, lowerbound, upperbound,
                  dimension, X_tr, X_te, Y_tr, Y_te):
    X = np.random.uniform(lowerbound, upperbound, (SearchAgents, dimension))
    fitness = np.zeros(SearchAgents)
    POP = [structure(cost=None, pre=None) for _ in range(SearchAgents)]

    for i in range(SearchAgents):
        fitness[i], POP[i].pre = cost_function(X[i], X_tr, X_te, Y_tr, Y_te)
        POP[i].cost = fitness[i]

    best_idx = np.argmin(fitness)
    Best_pos, Best_score, Best_pre = X[best_idx].copy(), fitness[best_idx], POP[best_idx].pre

    w_max, w_min, C, S, T = 0.9, 0.4, 1.5, 0.5, 0.3
    HO_curve = np.zeros(Max_iterations)

    for t in range(Max_iterations):
        w = w_max - (w_max - w_min) * (t / Max_iterations)
        for i in range(SearchAgents):
            candidates = [c for c in range(SearchAgents) if c != i]
            r1, r2 = np.random.choice(candidates, 2, replace=False)
            rand_val = np.random.rand()
            if rand_val < T:
                new_position = X[i] + levy_flight(dimension) * (X[r1] - X[r2])
            elif rand_val < T + S:
```

```
            phi = np.random.uniform(-1, 1, dimension)
            new_position = X[i] + w * phi * (Best_pos - X[i]) + \
                       np.random.rand() * (X[r1] - X[r2])
        else:
            A = 2 * np.random.rand() - 1
            new_position = X[i] + C * np.random.rand(dimension) * \
                       (A * (Best_pos - X[i]) + (1-A) * (X[r1] - X[r2]))
        new_position = np.clip(new_position, lowerbound, upperbound)
        new_fitness, new_pre = cost_function(new_position, X_tr, X_te, Y_tr, Y_te)
        if new_fitness < fitness[i]:
            X[i], fitness[i], POP[i].pre, POP[i].cost = new_position, new_fitness, new_pre,
new_fitness
            if new_fitness < Best_score:
                Best_score, Best_pos, Best_pre = new_fitness, new_position.copy(), new_pre

    if t % 20 == 0 and t > 0:
        n_restart = max(1, int(SearchAgents * 0.1))
        for idx in np.argsort(fitness)[-n_restart:]:
            X[idx] = np.random.uniform(lowerbound, upperbound, dimension)
            fitness[idx], POP[idx].pre = cost_function(X[idx], X_tr, X_te, Y_tr, Y_te)
            POP[idx].cost = fitness[idx]

    HO_curve[t] = Best_score
    print(f'Iteration {t+1}/{Max_iterations}, Best RMSE = {Best_score:.6f}')

  return Best_score, Best_pos, Best_pre, HO_curve
```

```
XX = np.loadtxt('input.txt')
Y = np.loadtxt('target.txt')
size_Y = len(Y)
size_tr = round(size_Y*0.8)
size_te = size_Y - size_tr
X_tr, X_te = XX[:size_tr], XX[size_tr:]
Y_tr = Y[:size_tr].reshape(size_tr)
Y_te = Y[size_tr:].reshape(size_te)

dimension, lowerbound, upperbound = 5, 1, 999
SearchAgents, Max_iterations = 50, 200

Best_score, Best_pos, Best_pre, HO_curve = APO_optimizer(
    SearchAgents, Max_iterations, lowerbound, upperbound, dimension,
    X_tr, X_te, Y_tr, Y_te)

ytr = Best_pre
N = len(Y)
MSE = np.mean((ytr - Y)**2)
RMSE = np.sqrt(MSE)
MAE = np.mean(np.abs(ytr - Y))
R2 = np.corrcoef(Y, ytr)[0, 1]**2
with np.errstate(divide='ignore', invalid='ignore'):
    MAPE = np.mean(np.abs((Y - ytr) / ytr)) * 100

print(f"RMSE: {RMSE:.6f}, MSE: {MSE:.6f}, MAE: {MAE:.6f}, R²: {R2:.6f}, MAPE: {MAPE:.2f}%")
```

---

8. Stacking.py — Stacking Ensemble (ADAR + LGBR)

python

```
import numpy as np
import pandas as pd
from sklearn.ensemble import AdaBoostRegressor, RandomForestRegressor
from sklearn.tree import DecisionTreeRegressor
from sklearn.model_selection import KFold, cross_val_predict
from sklearn.metrics import mean_squared_error, mean_absolute_error, r2_score
import lightgbm as lgb
import warnings
warnings.filterwarnings('ignore')

XX = np.loadtxt('input.txt')
Y = np.loadtxt('target.txt')
size_Y = len(Y)
size_tr = round(size_Y * 0.8)
size_te = size_Y - size_tr
X_tr, X_te = XX[:size_tr], XX[size_tr:]
Y_tr, Y_te = Y[:size_tr], Y[size_tr:]

weak_learner = DecisionTreeRegressor(max_depth=5)
adar = AdaBoostRegressor(estimator=weak_learner, n_estimators=100,
                         learning_rate=0.1, random_state=42)
lgbm = lgb.LGBMRegressor(n_estimators=100, learning_rate=0.1, max_depth=5,
                         num_leaves=31, subsample=0.8, colsample_bytree=0.8,
                         random_state=42, verbose=-1)
```

```
class StackingRegressor:
    def __init__(self, base_models, meta_model, n_folds=5):
        self.base_models = base_models
        self.meta_model = meta_model
        self.n_folds = n_folds
        self.base_models_trained = []

    def fit(self, X, y):
        from sklearn.base import clone
        self.base_models_trained = [clone(m) for m in self.base_models]
        for model in self.base_models_trained:
            model.fit(X, y)
        kf = KFold(n_splits=self.n_folds, shuffle=True, random_state=42)
        meta_features = np.column_stack([
            cross_val_predict(m, X, y, cv=kf) for m in self.base_models
        ])
        self.meta_model.fit(meta_features, y)
        return self

    def predict(self, X):
        base_predictions = np.column_stack([m.predict(X) for m in self.base_models_trained])
        return self.meta_model.predict(base_predictions)

stacking = StackingRegressor(
    base_models=[adar, lgbm],
    meta_model=RandomForestRegressor(n_estimators=100, random_state=42),
```

```
    n_folds=5
)
stacking.fit(X_tr, Y_tr)
adar.fit(X_tr, Y_tr)
lgbm.fit(X_tr, Y_tr)

y_pred_adar = adar.predict(X_te)
y_pred_lgbm = lgbm.predict(X_te)
y_pred_stacking = stacking.predict(X_te)

def evaluate_model(y_true, y_pred, name):
    mse = mean_squared_error(y_true, y_pred)
    rmse = np.sqrt(mse)
    mae = mean_absolute_error(y_true, y_pred)
    r2 = r2_score(y_true, y_pred)
    with np.errstate(divide='ignore', invalid='ignore'):
        mape = np.mean(np.abs((y_true - y_pred) / y_true)) * 100
    return {'Model': name, 'RMSE': rmse, 'MSE': mse, 'MAE': mae, 'R2': r2, 'MAPE': mape}

results = [evaluate_model(Y_te, y_pred_adar, 'ADAR'),
           evaluate_model(Y_te, y_pred_lgbm, 'LGBR'),
           evaluate_model(Y_te, y_pred_stacking, 'Stacking (ADAR + LGBR)')]
print(pd.DataFrame(results).to_string(index=False))

pd.DataFrame({'Actual': Y_te, 'ADAR_Pred': y_pred_adar,
              'LGBR_Pred': y_pred_lgbm, 'Stacking_Pred': y_pred_stacking
              }).to_csv('stacking_predictions.csv', index=False)
```

9. TOPSIS_2.py — TOPSIS Multi-Criteria Ranking

python

```
import numpy as np
import pandas as pd


def topsis(df, weights=None, criteria_type=None, normalize_method='vector'):
    if not isinstance(df, pd.DataFrame):
        raise ValueError("df must be a DataFrame")
    A = df.copy().astype(float)
    m, n = A.shape
    if A.isnull().values.any():
        A = A.fillna(A.mean())
    w = np.ones(n) / n if weights is None else np.array(weights, dtype=float)
    w = w / w.sum()
    crit = ['benefit'] * n if criteria_type is None else [c.lower() for c in criteria_type]

    X = A.values.copy()
    if normalize_method == 'vector':
        denom = np.sqrt((X ** 2).sum(axis=0))
        denom[denom == 0] = 1.0
        R = X / denom
    elif normalize_method == 'minmax':
        mn, mx = X.min(axis=0), X.max(axis=0)
        rng = mx - mn
        rng[rng == 0] = 1.0
        R = (X - mn) / rng
```

```
    V = R * w
    ideal = np.array([V[:, j].max() if crit[j] == 'benefit' else V[:, j].min() for j in range(n)])
    anti_ideal = np.array([V[:, j].min() if crit[j] == 'benefit' else V[:, j].max() for j in range(n)])

    S_plus = np.sqrt(((V - ideal) ** 2).sum(axis=1))
    S_minus = np.sqrt(((V - anti_ideal) ** 2).sum(axis=1))
    C = S_minus / (S_plus + S_minus)

    result_df = A.copy()
    result_df["distance_to_ideal"] = S_plus
    result_df["distance_to_negative_ideal"] = S_minus
    result_df["topsis_score"] = C
    result_df["rank"] = result_df["topsis_score"].rank(ascending=False).astype(int)
    return result_df.sort_values("rank")

data = {
    "RMSE": [1.013700381, 1.275900188, 1.404260114, 2.131630316],
    "R2":   [0.98478485,  0.968095862, 0.953438146, 0.929696818],
    "MSE":  [1.027588462, 1.627921291, 1.971946467, 4.543847802],
    "NSE":  [0.969839006, 0.952218494, 0.942120929, 0.866632439],
    "GR125":[0.729323308, 0.676691729, 0.511278195, 0.436090226],
}
df = pd.DataFrame(data, index=["Model_1", "Model_2", "Model_3", "Model_4"])
result = topsis(df, criteria_type=["cost", "benefit", "cost", "benefit", "benefit"])
print(result)
```

---

10. ANOVA_Statistical.py — Statistical Comparison of Models

python

```
import numpy as np
import pandas as pd
from scipy.stats import shapiro, levene, kruskal, f_oneway, ttest_ind, mannwhitneyu
from itertools import combinations
import warnings
warnings.filterwarnings('ignore')

pd.options.display.float_format = "{:.4f}".format

Data = np.loadtxt('Data.txt')
y_true = Data[:, 0]
y_preds = Data[:, 1:]

errors = pd.DataFrame({f'Model{i+1}': y_preds[:, i] - y_true for i in range(y_preds.shape[1])})

normality_results = []
for model in errors.columns:
    stat, p = shapiro(errors[model])
    normality_results.append([model, stat, p])

stat_levene, p_levene = levene(*[errors[model] for model in errors.columns])
all_normal = all(p > 0.05 for _, _, p in normality_results)
homogeneous_var = p_levene > 0.05

if all_normal and homogeneous_var:
```

```
    f_stat, p_val = f_oneway(*[errors[model] for model in errors.columns])
    results_summary = pd.DataFrame({"F-statistic": [f_stat], "p_value": [p_val]})
    if p_val < 0.05:
        model_names = errors.columns.tolist()
        n_comp = len(list(combinations(range(len(model_names)), 2)))
        comparisons = []
        for i, j in combinations(range(len(model_names)), 2):
            t_stat, p_raw = ttest_ind(errors[model_names[i]], errors[model_names[j]])
            comparisons.append([model_names[i], model_names[j], t_stat, p_raw,
                                min(p_raw * n_comp, 1.0)])
        posthoc = pd.DataFrame(comparisons, columns=['Model1', 'Model2', 't-stat', 'p_raw',
'p_corrected'])
        print(posthoc)
else:
    stat_kw, p_kw = kruskal(*[errors[model] for model in errors.columns])
    results_summary = pd.DataFrame({"H-statistic": [stat_kw], "p_value": [p_kw]})
    if p_kw < 0.05:
        model_names = errors.columns.tolist()
        n_comp = len(list(combinations(range(len(model_names)), 2)))
        comparisons = []
        for i, j in combinations(range(len(model_names)), 2):
            u_stat, p_raw = mannwhitneyu(errors[model_names[i]], errors[model_names[j]])
            comparisons.append([model_names[i], model_names[j], u_stat, p_raw,
                                min(p_raw * n_comp, 1.0)])
        posthoc = pd.DataFrame(comparisons, columns=['Model1', 'Model2', 'U-stat', 'p_raw',
'p_corrected'])
        print(posthoc)
```

```
print(results_summary)
```

---

11. wilcoxon.py — Wilcoxon Signed-Rank Test

python

```python
import numpy as np
import pandas as pd
from scipy.stats import wilcoxon

Y = np.loadtxt('target.txt')
Preds = np.loadtxt('Data.txt')

stat = np.zeros(len(Preds[0]))
p_value = np.zeros(len(Preds[0]))

for i in range(len(Preds[0])):
    stat[i], p_value[i] = wilcoxon(Y, Preds[:, i])

df = pd.DataFrame({
    'Model': np.arange(1, Preds.shape[1] + 1),
    'Stat': stat,
    'p_value': p_value
})
df['Rank_p'] = df['p_value'].rank(ascending=False, method='dense')
df['Rank_stat'] = df['Stat'].rank(ascending=True, method='dense')

print(df.sort_values('Rank_p'))
```
