# Supplementary material for: Automated machine-learning framework for predicting drug solubility in supercritical CO2 for sustainable process development
Source: Sci Rep. 2026 Jun 25;16:28594. doi: 10.1038/s41598-026-59449-z (PMC13575184; doi:10.1038/s41598-026-59449-z)
Supplement: Supplementary file 2 [file 41598_2026_59449_MOESM2_ESM.pdf]

S1: Gathered Solubility Dataset for the article

Temperature (K)	molecular weight (g/mol)	melting point (C)	Pressure (MPa)	Solubility (g/l) x 10
318	586.18	146	30	1.94
308	157.1	190	12	0.53
338	926.09	155	21	0.77
308	157.1	190	30	5.13
318	385.37	236	270	0.413
338	586.18	146	24	3.2
318	157.1	190	18	1.3
318	385.37	236	300	0.493
308	385.37	236	270	0.346
318	157.1	190	27	5.41
338	157.1	190	24	5.6
308	337.45	163.5	15	1.06
318	586.18	146	27	1.57
328	157.1	190	27	6.58
328	393.44	230	12	0.074
318	586.18	146	27	1.57
308	157.1	190	24	3.42
328	586.18	146	24	2.12
318	157.1	190	27	5.41
338	926.09	155	30	1.73
328	315.71	239	120	0.2
338	393.44	230	12	0.045
328	586.18	146	12	0.07
308	337.45	163.5	30	3.835
338	157.1	190	12	0.03
318	385.37	236	180	0.166
318	157.1	190	15	0.8
328	393.44	230	30	1.584
318	586.18	146	18	0.58
308	926.09	155	27	1.45
338	393.44	230	30	1.68
318	926.09	155	21	1.02
338	337.45	163.5	21	2.177
318	157.1	190	12	0.37
338	393.44	230	24	1.049
328	157.1	190	21	3.21
328	393.44	230	18	0.368
318	157.1	190	30	6.48

328	586.18	146	27	2.94
308	586.18	146	15	0.24
328	586.18	146	24	2.12
318	157.1	190	30	6.48
328	315.71	239	210	1.91
338	385.37	236	300	0.665
338	157.1	190	24	5.6
338	315.71	239	210	1.83
318	926.09	155	24	1.23
328	385.37	236	270	0.473
338	157.1	190	15	0.37
338	157.1	190	18	1.32
318	393.44	230	27	1.259
338	586.18	146	12	0.04
338	586.18	146	15	0.16
318	315.71	239	300	3.76
308	385.37	236	120	0.042
308	337.45	163.5	24	2.377
318	157.1	190	18	1.3
308	926.09	155	18	0.92
328	157.1	190	12	0.08
308	586.18	146	24	0.61
308	586.18	146	30	0.88
308	385.37	236	210	0.216
308	385.37	236	150	0.097
318	385.37	236	240	0.34
328	157.1	190	18	1.39
328	157.1	190	12	0.08
308	385.37	236	300	0.42
318	157.1	190	24	4.29
338	337.45	163.5	24	3.189
318	337.45	163.5	21	1.895
308	315.71	239	150	1.41
318	337.45	163.5	27	3.445
338	385.37	236	150	0.083
308	157.1	190	21	2.31
308	157.1	190	24	3.42
318	393.44	230	21	0.73
308	586.18	146	12	0.13
328	315.71	239	270	3.3
308	586.18	146	12	0.13

318	586.18	146	24	1.22
308	586.18	146	27	0.74
308	337.45	163.5	21	1.857
318	337.45	163.5	15	0.746
338	385.37	236	210	0.336
328	337.45	163.5	12	0.12
338	586.18	146	18	0.77
308	337.45	163.5	12	0.77
338	157.1	190	21	3.92
308	315.71	239	120	1.09
338	157.1	190	15	0.37
338	337.45	163.5	30	5.153
308	315.71	239	300	3.07
318	926.09	155	12	0.27
338	157.1	190	12	0.03
328	586.18	146	18	0.76
328	393.44	230	27	1.237
318	157.1	190	21	2.72
338	337.45	163.5	12	0.046
328	157.1	190	15	0.6
308	393.44	230	30	1.289
308	393.44	230	12	0.279
308	393.44	230	15	0.395
338	393.44	230	18	0.26
328	393.44	230	24	1.032
338	315.71	239	150	0.56
328	157.1	190	21	3.21
308	393.44	230	27	1.075
308	926.09	155	21	1.15
318	157.1	190	15	0.8
308	586.18	146	18	0.36
318	315.71	239	210	2.01
328	385.37	236	210	0.277
308	393.44	230	18	0.609
328	157.1	190	18	1.39
328	157.1	190	30	7.65
338	157.1	190	21	3.92
328	315.71	239	240	2.52
318	586.18	146	12	0.1
328	337.45	163.5	30	4.714
338	926.09	155	18	0.45

318	393.44	230	18	0.443
328	586.18	146	27	2.94
328	926.09	155	24	1.15
328	157.1	190	15	0.6
318	157.1	190	12	0.37
318	926.09	155	18	0.75
328	586.18	146	30	3.83
318	586.18	146	21	0.89
318	586.18	146	18	0.58
328	337.45	163.5	18	1.325
338	586.18	146	18	0.77
308	385.37	236	240	0.27
328	586.18	146	18	0.76
338	315.71	239	240	2.56
328	337.45	163.5	24	2.742
308	337.45	163.5	18	1.553
308	926.09	155	30	1.61
308	315.71	239	240	2.35
308	586.18	146	15	0.24
308	926.09	155	15	0.69
318	393.44	230	24	1.022
338	337.45	163.5	27	4.294
308	586.18	146	27	0.74
308	157.1	190	27	4.09
338	385.37	236	180	0.21
338	926.09	155	12	0.03
308	586.18	146	30	0.88
328	385.37	236	240	0.384
328	586.18	146	21	1.38
338	393.44	230	27	1.292
338	157.1	190	27	7.57
328	315.71	239	300	4.19
338	157.1	190	27	7.57
308	157.1	190	12	0.53
308	157.1	190	30	5.13
338	586.18	146	24	3.2
328	385.37	236	120	0.028
328	337.45	163.5	27	3.908
338	385.37	236	240	0.433
338	385.37	236	270	0.562
338	315.71	239	270	3.39

308	385.37	236	180	0.144
328	315.71	239	150	0.83
328	385.37	236	180	0.18
328	926.09	155	21	0.89
308	315.71	239	180	1.66
328	926.09	155	30	1.71
308	315.71	239	210	2.03
308	393.44	230	24	0.918
308	315.71	239	270	2.76
308	337.45	163.5	27	3.017
338	586.18	146	27	4.73
338	586.18	146	30	6.12
308	586.18	146	21	0.48
338	337.45	163.5	15	0.329
308	157.1	190	15	0.87
308	586.18	146	24	0.61
328	393.44	230	21	0.673
338	586.18	146	27	4.73
328	926.09	155	15	0.37
318	385.37	236	120	0.034
328	586.18	146	21	1.38
318	586.18	146	21	0.89
338	926.09	155	27	1.53
318	315.71	239	270	2.95
318	315.71	239	180	1.54
328	393.44	230	15	0.175
328	157.1	190	27	6.58
328	337.45	163.5	21	2
318	586.18	146	12	0.1
328	586.18	146	15	0.2
328	385.37	236	300	0.551
338	926.09	155	15	0.22
308	586.18	146	18	0.36
328	157.1	190	30	7.65
318	385.37	236	150	0.094
338	586.18	146	12	0.04
328	586.18	146	15	0.2
308	157.1	190	18	1.44
318	385.37	236	210	0.237
318	926.09	155	15	0.53
308	157.1	190	18	1.44

328	926.09	155	18	0.64
318	926.09	155	27	1.48
338	315.71	239	120	0.11
318	586.18	146	30	1.94
328	315.71	239	180	1.39
338	157.1	190	30	9.05
318	315.71	239	120	0.69
328	586.18	146	12	0.07
338	157.1	190	18	1.32
338	586.18	146	15	0.16
308	157.1	190	27	4.09
318	315.71	239	240	2.44
318	315.71	239	150	1.13
328	157.1	190	24	4.75
318	586.18	146	24	1.22
318	157.1	190	24	4.29
318	586.18	146	15	0.22
318	157.1	190	21	2.72
308	926.09	155	24	1.28
318	337.45	163.5	24	2.557
308	393.44	230	21	0.764
338	157.1	190	30	9.05
318	393.44	230	15	0.296
318	926.09	155	30	1.69
328	385.37	236	150	0.09
328	586.18	146	30	3.83
338	315.71	239	300	4.22
318	337.45	163.5	12	0.351
338	385.37	236	120	0.019
338	315.71	239	180	1.15
328	157.1	190	24	4.75
338	586.18	146	30	6.12
318	586.18	146	15	0.22
318	393.44	230	30	1.494
328	926.09	155	12	0.12
308	586.18	146	21	0.48
308	157.1	190	21	2.31
338	393.44	230	21	0.591
338	393.44	230	15	0.119
318	393.44	230	12	0.161
338	337.45	163.5	18	1.23

318	337.45	163.5	30	4.358
318	337.45	163.5	18	1.399
308	926.09	155	12	0.45
328	337.45	163.5	15	0.542
338	926.09	155	24	1.11
338	586.18	146	21	1.81
338	586.18	146	21	1.81
308	157.1	190	15	0.87
328	926.09	155	27	1.53
